# A Time-Varying Mixture Integer-Valued Threshold Autoregressive Process Driven by Explanatory Variables

**DOI:** 10.3390/e26020140

**Published:** 2024-02-04

**Authors:** Danshu Sheng, Dehui Wang, Jie Zhang, Xinyang Wang, Yiran Zhai

**Affiliations:** 1 School of Mathematics and Statistics, Liaoning University, Shenyang 110031, China; 2 School of Mathematics and Statistics, Changchun University of Technology, Changchun 130012, China; 3 State Grid Jilin Electric Power Company Limited Information and Telecommunication Company, Changchun 132400, China

**Keywords:** threshold integer-valued autoregressive models, mixture thinning operator, parameter estimation, Wald test, explanatory variables

## Abstract

In this paper, a time-varying first-order mixture integer-valued threshold autoregressive process driven by explanatory variables is introduced. The basic probabilistic and statistical properties of this model are studied in depth. We proceed to derive estimators using the conditional least squares (CLS) and conditional maximum likelihood (CML) methods, while also establishing the asymptotic properties of the CLS estimator. Furthermore, we employed the CLS and CML score functions to infer the threshold parameter. Additionally, three test statistics to detect the existence of the piecewise structure and explanatory variables were utilized. To support our findings, we conducted simulation studies and applied our model to two applications concerning the daily stock trading volumes of VOW.

## 1. Introduction

An integer-valued time series represents count data reflecting the states of a particular phenomenon at different time points. It finds widespread applications across various real-world domains. For instance, in the field of economics, Ref. [1] employed the integer-valued moving average model to describe the number of transactions in intra-day stock data. In industrial contexts, Ref. [2] utilized the compound Poisson integer-valued autoregressive (INAR) model to characterize the count of workers in the heavy manufacturing industry receiving benefits due to burn-related injuries. Within the realm of insurance actuarial studies, Ref. [3] explored an extension of the classical discrete-time risk model, incorporating an INAR(1) process to capture temporal dependence among claim counts. A straightforward approach in the modeling and analyzing of count time series involves creating an integer-valued autoregressive model by using thinning operators. Ever since [4] introduced the first INAR(1) time series model, which relied on the binomial thinning operator [5], it has become a prevalent method to model INAR-type models utilizing various thinning operators (see [6,7,8,9,10], among others). This approach has been extensively applied across diverse fields such as epidemiology, social sciences, economics, life sciences, and more.

Even though INAR-type models are commonly used in practical applications, they often fall short when confronted with nonlinear phenomena. For instance, researchers in the field of epidemiology, as exemplified by [11], have detected temporal fluctuations in the incidence rate of epidemics. Therefore, trying to represent such data with an INAR-type model may not be the most-appropriate approach. Furthermore, time series data frequently undergo sudden changes that can either temporarily or permanently disrupt their dynamics. In such situations, the conventional INAR model may also prove inadequate in delivering an accurate fit. To address the nonlinear aspects of integer-valued time series data, Ref. [12] introduced the integer-valued self-exciting threshold autoregressive (SETINAR(2,1)) process, which relies on the binomial thinning operator (“∘”); Ref. [13] presented the self-excited threshold Poisson autoregressive (SETPAR) model and applied it to analyze major global earthquake data; Ref. [14] proposed a basic self-exciting threshold binomial AR(1) model (SETBAR(1)) with values across a finite range of counts; Ref. [15] investigated an integer-valued threshold autoregressive process (NBTINAR(1)) based on the negative binomial thinning operator (“∗”) and applied it to analyze the annual counts of major earthquakes with a magnitude of 7 or above from 1900 to 2015. In a comprehensive review, Ref. [16] surveyed threshold models for integer-valued time series with an infinite range and introduced two novel models tailored to cases with a finite range of values. In the latest research, Ref. [17] pointed out that employing different operators before and after the threshold can enhance the model’s ability to explain a wider range of phenomena. As a result, she has proposed the following threshold autoregressive model using the mixed operators.

$$X_{t}=\left\{\begin{array}{cc} \alpha_{1}\circ X_{t-1}+Z_{1,t}, & X_{t-1}\leq \;r\\ \alpha_{2}*X_{t-1}+Z_{2,t}, & X_{t-1}>r; \end{array}\right.$$

where $\left\{Z_{1,t}\right\}$ and $\left\{Z_{2,t}\right\}$ are sequences of i.i.d. Poisson and Geometric distributed random variables, respectively. However, it is worth noting that the use of constant autoregressive coefficients in this model ignores the effect of exogenous variables on the observed data. For example, denote the daily trading volume of a specific stock as $X_{t}$. Clearly, in practice, its autoregressive coefficient is often not static and is often subject to some external factors related to change over time, such as: market factors: the overall volatility of the stock market can impact the volatility of individual stocks; industry factors: specific events or trends within an industry can also affect stock price fluctuations; interest rates and monetary policy: changes in interest rates and monetary policies can have a wide-ranging impact on the stock market; political and geopolitical factors: political events, elections, international relations, and geopolitical tensions can introduce uncertainty and volatility to the stock market. emotional and investor behavior: investor sentiment and behavior can significantly influence stock price movements, among other factors.

Inspired by the above discussion and learning the method of constructing models driven by explanatory variables (see [18,19], just to name a few), in this paper, we propose a first-order time-varying mixture thinning integer-valued threshold autoregressive (TVMTTINAR(1)) process driven by explanatory variables. For this, the definition of the TVMTTINAR(1) model is given, and the statistical inference for the proposed model is studied. Furthermore, considering that verifying the existence of a piecewise structure and explanatory variables is key to model construction, we propose three kinds of test statistics. Finally, from the simulation and two applications, we can also see that our proposed model is very competitive.

The organization of this paper is as follows. Section 2 gives the definition of the proposed model, and some properties are also investigated. In Section 3, the estimates of the model parameters are derived by using the conditional least squares (CLS) and conditional maximum likelihood (CML) methods. Three test statistics are also constructed to test the existence of the piecewise structure and explanatory variables, respectively. Some simulation studies are carried out to investigate the performances of the proposed estimates and test statistics in Section 4. Two real data examples are given in Section 5. Some concluding remarks are given in Section 6. All proofs are postponed to Appendix A.

## 2. The First-Order Time-Varying Mixture Thinning Integer-Valued Threshold Autoregressive Model

We first introduce the definition of the TVMTTINAR(1) process. Furthermore, we investigate the statistical properties of the proposed model.

**Definition** **1.** *The process $\left\{X_{t}\right\}$ is called the TVMTTINAR(1) process if $X_{t}$ follows the recursion:*

(1) $$\begin{array}{c} X_{t}=\left\{\begin{array}{cc} \phi_{1,t}\circ X_{t-1}+\varepsilon_{t}, & X_{t-1}\leq r\\ \phi_{2,t}*X_{t-1}+\varepsilon_{t}, & X_{t-1}>r, \end{array}\right. \end{array}$$

*or*

(2) $$\begin{array}{c} X_{t}=\left\{\begin{array}{cc} \phi_{2,t}*X_{t-1}+\varepsilon_{t}, & X_{t-1}\leq r\\ \phi_{1,t}\circ X_{t-1}+\varepsilon_{t}, & X_{t-1}>r. \end{array}\right. \end{array}$$

*For convenience, we write the above two models by the symbol R as follows:*

(3) $$X_{t}=\left(\phi_{1,t}\circ X_{t-1}\right)I_{1,t}^{R}+\left(\phi_{2,t}*X_{t-1}\right)I_{2,t}^{R}+\varepsilon_{t},\;\;t\in Z,$$

*where:*

1. *$I_{1,t}^{R}=\left\{\begin{array}{c} I\{X_{t-1}\leq r\},R=0,\\ I\{X_{t-1}>r\},R=1, \end{array}\right.$ and $I_{2,t}^{R}=1-I_{1,t}^{R}=\left\{\begin{array}{c} I\{X_{t-1}>r\},R=0,\\ I\{X_{t-1}\leq r\},R=1; \end{array}\right.$ That is, R=0 indicates that TVMTTINAR(1) represents the process (1).*
2. *For fixed i∈1,2, $\phi_{i,t}\in \left(0,1\right)$*
$$\log\left(\frac{\phi_{i,t}}{1-\phi_{i,t}}\right)=Z_{t}^{⊤}\beta_{i},$$
  *where $\beta_{i}={\left(\beta_{i,0},\beta_{i,1},\ldots ,\beta_{i,q}\right)}^{⊤}$ are the regression coefficients, ${\left\{Z_{t}:={\left(1,Z_{1,t},\ldots ,Z_{q,t}\right)}^{⊤}\right\}}_{t\in Z}$ is a sequence of stationary, weakly dependent, and observable explanatory variables with a constant mean vector and covariance matrix. For fixed t, $Z_{t}$ is assumed to be independent of ${\left\{X_{t-l}\right\}}_{l\geq 1}$.*
3. *The binomial thinning operator “∘”, proposed by [5], is defined as $\phi_{1}\circ X=\sum_{i=1}^{X}B_{i}$, where ϕ∈(0,1), $\left\{B_{i}\right\}$ is a sequence of i.i.d. Bernoulli random variables satisfying $P\left(B_{i}=1\right)=1-P\left(B_{i}=0\right)=\phi =$ and $B_{i}$ is independent of X.*
4. *The negative binomial thinning operator “∗”, proposed by [20], is defined as $\phi *X=\sum_{i=1}^{X}W_{i}$, where ϕ∈(0,1), $\left\{W_{i}\right\}$ is a sequence of i.i.d. Geometric random variables with parameter $\frac{\phi }{1+\phi_{2}}$ and $W_{i}$ is independent of X.*
5. *$\left\{\varepsilon_{t}\right\}$ is a sequence of i.i.d. Poisson distributed random variables with mean λ. For fixed t, $\varepsilon_{t}$ is assumed to be independent of $\phi \circ X_{t-1}$, $\phi *X_{t-1}$, and $X_{t-l}$ for all l≥1.*

In contrast to the common SETINAR-type model, the TVMTTINAR model does not require $\beta_{1}=\beta_{2}$. This is mainly due to the existence of mixed thinning operators. Even when $\beta_{1}=\beta_{2}$, there is a piecewise structure. However, this is a small probability event, and the model inference problem in this case is not specially considered in this paper. In addition, in practical applications, we can usually choose which of the two TVMTTINAR(1) models (R=0 and R=1) is more applicable based on criteria such as the AIC and BIC. We will conduct a specific analysis through data examples in Section 5.

Next, we are ready to state that there exists the strict stationarity and ergodicity of the VTMTTINAR(1) process. Note that, under the assumption that $\beta_{i}$ satisfies $\sup_{Z\in R^{q+1}}\left|\beta_{i}Z\right|<\infty$ for i=1,2, there is

$$\phi_{i,t}=\frac{\exp(Z_{t}^{⊤}\beta_{i})}{1+\exp(Z_{t}^{⊤}\beta_{i})}\in \left(0,1\right),\forall t.$$

Thereby, similar to the method in [19], it is easy to verify that Theorem 3.1 in [21] holds, and the TVMTTINAR(1) process is a strictly stationary and ergodic Markov chain. Moreover, its transition probabilities are given by

(4) $$\begin{array}{cc} P(z_{t},x_{t-1},x_{t}) & =P(X_{t}=x_{t}|X_{t-1}=x_{t-1},Z_{t}=z_{t})\\ \; & =P\left(\phi_{1,t}\circ X_{t-1}I_{1,t}^{R}+\phi_{2,t}*X_{t-1}I_{2,t}^{R}+\varepsilon_{t}=x_{t}|X_{t-1}=x_{t-1},Z_{t}=z_{t}\right)\\ \; & =p\left(x_{t-1},x_{t},\phi_{1,t},\lambda \right)I_{1,t}^{R}+p\left(x_{t-1},x_{t},\phi_{2,t},\lambda \right)I_{2,t}^{R}\\ \; & =\sum_{i=1}^{2}p\left(x_{t-1},x_{t},\phi_{i,t}I_{i,t}^{R},\lambda \right), \end{array}$$

where

(5) p(xt−1,xt,ϕ1,tI1,tR,λ)=I1,tR∑m=0min(xt−1,xt)xt−1me−λλxt−m(xt−m)!ϕ1,tm(1−ϕ1,t)xt−1−m,p(xt−1,xt,ϕ2,tI2,tR,λ)=I2,tR∑m=0xtΓ(xt−1+m)Γ(xt−1)Γ(m+1)ϕ2,tm(1+ϕ2,t)xt−1+me−λλxt−m(xt−m)!.

Since the existence of the first four moments under observable explanatory variables is a necessary condition for deriving the asymptotic properties of the parameter estimation in Section 3, we then give the following proposition.

**Proposition** **1.** *Let* $\left\{X_{t}\right\}$ *be the process defined by Definition 1. Then, the first four conditional moments are bounded, that is* $E(X_{t}^{k}|Z_{0},\ldots ,Z_{t})<\infty$ *for* k=1,2,3,4.

Next, we consider the moments and conditional moments of TVMTTINAR(1). For the simplicity of the notation, we denote $E\left(I_{1,t}^{R}\right)=p_{1}$, $E\left(I_{2,t}^{R}\right)=p_{2}=1-p_{1}$, $\mu_{1}:=E\left(X_{t}|X_{t}\leq r\right)$, $\mu_{2}:=E\left(X_{t}|X_{t}>r\right)$, $\mu_{\phi_{1}}^{\left(h\right)}:=E\left(\frac{\exp(Z_{t+h}^{⊤}\beta_{1})}{1+\exp(Z_{t+h}^{⊤}\beta_{1})}|X_{t+h-1}\leq r\right)$, $\mu_{\phi_{2}}^{\left(h\right)}:=E\left(\frac{\exp(Z_{t+h}^{⊤}\beta_{2})}{1+\exp(Z_{t+h}^{⊤}\beta_{2})}|X_{t+h-1}>r\right)$, $\phi_{i}:=E\left(\frac{\exp(Z_{t}^{⊤}\beta_{i})}{1+\exp(Z_{t}^{⊤}\beta_{i})}\right)\;\left(i=1,2\right)$, $\sigma_{\phi_{i}}^{2}:=\mathrm{Var}\left(\frac{\exp(Z_{t}^{⊤}\beta_{i})}{1+\exp(Z_{t}^{⊤}\beta_{i})}\right)\;\left(i=1,2\right)$, $\sigma_{1}^{2}:=\mathrm{Var}\left(X_{t}|X_{t}\leq r\right)$, $\sigma_{2}^{2}:=\mathrm{Var}\left(X_{t}|X_{t}>r\right)$, $\gamma_{h}^{\left(1\right)}:=\mathrm{Cov}\left(X_{t},X_{t+h}|X_{t+h}\leq r\right)$, $\gamma_{h}^{\left(2\right)}:=\mathrm{Cov}\left(X_{t},X_{t+h}|X_{t+h}>r\right),$ where $\gamma_{0}^{\left(i\right)}=\left[\left(\sigma_{i}^{2}+\mu_{i}^{2}\right)-\mu_{i}E\left(X_{t}\right)\right],i=1,2.$

The conditional mean and variance for the TVMTTINAR(1) model can be given by

$$\begin{array}{cc} \; & E\left(X_{t}|X_{t-1},Z_{t}\right)=\sum_{i=1}^{2}\frac{\exp(Z_{t}^{⊤}\beta_{i})}{1+\exp(Z_{t}^{⊤}\beta_{i})}X_{t-1}I_{i,t}^{R}+\lambda ,\\ \; & E\left(X_{t}|Z_{t}\right)=\sum_{i=1}^{2}\frac{\exp(Z_{t}^{⊤}\beta_{i})}{1+\exp(Z_{t}^{⊤}\beta_{i})}p_{i}\mu_{i}+\lambda ,\\ \; & \mathrm{Var}\left(X_{t}|X_{t-1},Z_{t}\right)=\frac{\exp(Z_{t}^{⊤}\beta_{1})}{{\left[1+\exp\left(Z_{t}^{⊤}\beta_{1}\right)\right]}^{2}}X_{t-1}I_{1,t}^{R}+\frac{\exp\left(Z_{t}^{⊤}\beta_{2}\right)\left[1+2\exp\left(Z_{t}^{⊤}\beta_{2}\right)\right]}{{\left[1+\exp\left(Z_{t}^{⊤}\beta_{2}\right)\right]}^{2}}X_{t-1}I_{1,t}^{R}+\lambda ,\\ \; & \mathrm{Var}\left(X_{t}|Z_{t}\right)=\sum_{i=1}^{2}\left\{{\left[\frac{\exp(Z_{t}^{⊤}\beta_{i})}{1+\exp(Z_{t}^{⊤}\beta_{i})}\right]}^{2}\left[p_{i}\left(\sigma_{i}^{2}+\mu_{i}^{2}\right)-p_{i}^{2}\mu_{i}^{2}\right]\right\}+\frac{\exp(Z_{t}^{⊤}\beta_{1})}{{\left[1+\exp\left(Z_{t}^{⊤}\beta_{1}\right)\right]}^{2}}p_{1}\mu_{1}\\ \; & \;\;\;\;\;\;\;\;\;\;\;\;\;\;\;\;\;\;+\frac{\exp\left(Z_{t}^{⊤}\beta_{2}\right)\left[1+2\exp\left(Z_{t}^{⊤}\beta_{2}\right)\right]}{{\left[1+\exp\left(Z_{t}^{⊤}\beta_{2}\right)\right]}^{2}}p_{2}\mu_{2}-2\prod_{i=1}^{2}\frac{\exp(Z_{t}^{⊤}\beta_{i})}{1+\exp(Z_{t}^{⊤}\beta_{i})}p_{i}\mu_{i}+\lambda . \end{array}$$

The unconditional expressions for the marginal mean and variance of the TVMTTINAR(1) model are

$$\begin{array}{cc} \; & E\left(X_{t}\right)=\sum_{i=1}^{2}p_{i}\phi_{i}\mu_{i}+\lambda ,\\ \; & \mathrm{Var}\left(X_{t}\right)=\sum_{i=1}^{2}\left\{\phi_{i}^{2}\left[p_{i}\left(\sigma_{i}^{2}+\mu_{i}^{2}\right)-p_{i}^{2}\mu_{i}^{2}\right]+p_{i}\sigma_{\phi_{i}}^{2}\left(\sigma_{i}^{2}+\mu_{i}^{2}\right)\right\}+p_{1}\mu_{1}\left(\phi_{1}-\sigma_{\phi_{1}}^{2}-\phi_{1}^{2}\right)\\ \; & \;\;\;\;\;\;\;\;\;\;\;\;\;+p_{2}\mu_{2}\left(\phi_{2}+\sigma_{\phi_{2}}^{2}+\phi_{2}^{2}\right)-2p_{1}p_{2}\phi_{1}\phi_{2}\mu_{1}\mu_{2}+\lambda . \end{array}$$

Then, we have that the autocovariance function and autocorrelation function (ACF):

$$\begin{array}{cc} \; & \mathrm{Cov}\left(X_{t},X_{t+h}|Z_{t+1},\ldots ,Z_{t+h}\right)=\sum_{i=1}^{2}\frac{\exp(Z_{t+h}^{⊤}\beta_{i})}{1+\exp(Z_{t+h}^{⊤}\beta_{i})}p_{i}\gamma_{h-1}^{\left(i\right)},\\ \; & \mathrm{Cov}\left(X_{t},X_{t+h}\right)=\sum_{i=1}^{2}\mu_{\phi_{i}}^{\left(h\right)}p_{i}\gamma_{h-1}^{\left(i\right)},\\ \; & \rho \left(h\right):=\mathrm{Corr}\left(X_{t},X_{t+h}\right)=\left[\sum_{i=1}^{2}\mu_{\phi_{i}}^{\left(h\right)}p_{i}\gamma_{h-1}^{\left(i\right)}\right]\backslash \mathrm{Var}\left(X_{t}\right) \end{array}$$

## 3. Parameters’ Estimation and Testing

Suppose we have a series of observations ${\left\{X_{t}\right\}}_{t=1}^{n}$ generated from the TVMTTINAR(1) process. Denote $\theta ={\left(\beta_{1}^{⊤},\beta_{2}^{⊤},\lambda \right)}^{⊤}$ as the parameter vector under the known threshold parameter *r*, and $\eta ={\left(\beta_{1}^{⊤},\beta_{2}^{⊤},\lambda ,r\right)}^{⊤}$ as the parameter vector under the unknown *r* case. Their parameter spaces are

$$\Theta_{\theta }=\left\{\theta \in R^{q+1}\times R^{q+1}\times \left(0,+\infty \right)\right\},$$

$$\Theta_{\eta }=\left\{\eta \in R^{q+1}\times R^{q+1}\times \left(0,+\infty \right)\times N\right\}.$$

Furthermore, suppose the true values $\theta_{0}={\left(\beta_{1,0}^{⊤},\beta_{2,0}^{⊤},\lambda_{0}\right)}^{⊤}$ and $\eta_{0}={\left(\beta_{1,0}^{⊤},\beta_{2,0}^{⊤},\lambda_{0},r_{0}\right)}^{⊤}$ of $\theta_{0}$ and $\eta_{0}$ are the interior points of $\Theta_{\theta }$ and $\Theta_{\eta }$, respectively. In this section, we mainly implement parameter estimation based on two different approaches, namely the conditional least squares (CLS) and conditional maximum likelihood (CML) methods. The objective function is not differentiable with respect to the threshold variable *r* since *r* is an integer. Therefore, we firstly propose solutions to estimate θ under the assumption that the threshold variable *r* is known. Later, in Section 3.3, we turn to estimating the threshold variable *r* based on the estimation methods mentioned before. All the proofs are presented in Appendix A.

### 3.1. Conditional Least Squares Estimation

Denote

$$g\left(\theta ,X_{t-1},Z_{t}\right)=E\left(X_{t}|X_{t-1},Z_{t}\right)=\frac{\exp(Z_{t}^{⊤}\beta_{1})}{1+\exp(Z_{t}^{⊤}\beta_{1})}X_{t-1}I_{1,t}^{R}+\frac{\exp(Z_{t}^{⊤}\beta_{2})}{1+\exp(Z_{t}^{⊤}\beta_{2})}X_{t-1}I_{2,t}^{R}+\lambda ,$$

$$\begin{array}{cc} Q(\theta ) & :=\sum_{t=1}^{n}{\left(X_{t}-g\left(\theta ,X_{t-1},Z_{t}\right)\right)}^{2}=\sum_{t=1}^{n}U_{t}^{2}\left(\theta \right), \end{array}$$

where

$$U_{t}\left(\theta \right)=X_{t}-\frac{\exp(Z_{t}^{⊤}\beta_{1})}{1+\exp(Z_{t}^{⊤}\beta_{1})}X_{t-1}I_{1,t}^{R}-\frac{\exp(Z_{t}^{⊤}\beta_{2})}{1+\exp(Z_{t}^{⊤}\beta_{2})}X_{t-1}I_{2,t}^{R}-\lambda .$$

Then, the CLS estimator ${\hat{\theta }}_{CLS}:={\left({\hat{\beta }}_{1,CLS}^{⊤},{\hat{\beta }}_{2,CLS}^{⊤},{\hat{\lambda }}_{CLS}\right)}^{T}$ of θ is obtained by minimizing the sum of the squared deviations, that is

(6) $$\begin{array}{c} {\hat{\theta }}_{CLS}=\arg\min_{\theta \in \Theta_{\theta }}Q\left(\theta \right). \end{array}$$

Since the TVMTTINAR(1) model is stationary and ergodic and the first four moments are bounded, then using the Taylor expansion and the martingale central limit theorem, the following theorem about the consistency and asymptotic normality of the parameter estimator can be obtained. The detailed proof is presented in Appendix A.

**Theorem** **1.** *Let* $\left\{X_{t}\right\}$ *be a TVMTTINAR(1) process. Then, the CLS estimator* ${\hat{\theta }}_{CLS}$ *is consistent and has the asymptotic distribution:*

(7) $$\begin{array}{c} \sqrt{n}\left({\hat{\theta }}_{CLS}-\theta_{0}\right)\overset{d}{⟶}N\left(0,V^{-1}WV^{-1}\right), \end{array}$$

*where* V *and* W *are square matrices of order* 2q+3 *with the* jkth *element given by*

$$V_{jk}:=E{\left(\frac{\partial }{\partial \theta_{j}}g\left(\theta ,X_{t-1},Z_{t}\right)\frac{\partial }{\partial \theta_{k}}g\left(\theta ,X_{t-1},Z_{t}\right)\right)}_{\theta_{0}},$$

$$W_{jk}:=E{\left(U_{t}^{2}\left(\theta \right)\frac{\partial }{\partial \theta_{j}}g\left(\theta ,X_{t-1},Z_{t}\right)\frac{\partial }{\partial \theta_{k}}g\left(\theta ,X_{t-1},Z_{t}\right)\right)}_{\theta_{0}}.$$

### 3.2. Conditional Maximum Likelihood Estimation

For a fixed value of $x_{0}$, the conditional log-likelihood function for the TVMTTINAR(1) model can be written as

$$\begin{array}{c} L\left(\theta \right):=\sum_{t=1}^{n}\ell_{t}\left(\theta \right)=\sum_{t=1}^{n}\log P\left(z_{t},X_{t-1},X_{t}\right) \end{array}$$

where $P(z_{t},X_{t-1},X_{t})$ is the transition probabilities defined in (4). The CML estimator ${\hat{\theta }}_{CML}:={\left({\hat{\beta }}_{1,CML}^{⊤},{\hat{\beta }}_{2,CML}^{⊤},{\hat{\lambda }}_{CML}\right)}^{T}$ of θ is obtained by maximizing the conditional log-likelihood function, that is

(8) $$\begin{array}{c} {\hat{\theta }}_{CML}=\arg\max_{\theta \in \Theta_{\theta }}L\left(\theta \right). \end{array}$$

Since $\phi_{i,t}$ is nonlinear with respect to $\beta_{i,j}$ for arbitrary i=1,2 and j=0,1,…,q, there is no closed-form expressions for the CLS and CML estimators. The numerical solutions can be solved by the MATLAB(2021b) function “fmincon” or the R(4.2.1) function “optim”. The implementation details and performance are discussed in Section 4.

**Theorem** **2.** *Let* $\left\{X_{t}\right\}$ *be a TVMTTINAR(1) process. Assume that the function* $E[\ell_{t}\left(\theta \right)]$ *has a unique maximizer in the compact parameter space* **Θ**, $E{\left(\frac{1}{n}\frac{\partial^{2}\ell \left(\theta \right)}{\partial \theta \partial \theta^{⊤}}\right)}_{\theta_{0}}$ *is a nonsingular matrix, and for a neighborhood of* $\theta_{0}$*, say* $N(\theta_{0})$. *For any* i,j,k=1,…,2q+3, *there is*

$$\bar{\lim_{n\to \infty }}\underset{\theta \in N(\theta_{0})}{\sup}|\frac{\partial^{3}\ell_{t}\left(\theta \right)}{\partial \theta_{i}\partial \theta_{j}\partial \theta_{k}}|<\infty .$$

*Then, the CML estimator* ${\hat{\theta }}_{CML}$ *is consistent and has the asymptotic distribution:*

(9) $$\begin{array}{c} \sqrt{n}\left({\hat{\theta }}_{CML}-\theta_{0}\right)\overset{d}{⟶}N\left(0,J^{-1}\left(\theta_{0}\right)I\left(\theta_{0}\right)J^{-1}\left(\theta_{0}\right)\right), \end{array}$$

*as* n→∞, *where* $I\left(\theta_{0}\right)=E{\left[\frac{\partial \ell_{t}\left(\theta \right)}{\partial \theta }\frac{\partial \ell_{t}\left(\theta \right)}{\partial \theta^{⊤}}\right]}_{\theta_{0}}$, $J\left(\theta_{0}\right)=E{\left(\frac{\partial^{2}\ell_{t}\left(\theta \right)}{\partial \theta \partial \theta^{⊤}}\right)}_{\theta_{0}}$.

### 3.3. Inference Methods for Threshold r

In this section, we concentrate on the estimation of threshold variable *r*. Since *r* is an integer, different from the continuous-type threshold models, the integer-type threshold models usually use a one-by-one search on a fixed interval $\left[\underline{r},\bar{r}\right]$ to make the loss function optimal to obtain the threshold estimator. In applications, typically, the empirical 10th and 90th quantile value of the sample as $\underline{r}$ and $\bar{r}$ is used, respectively. The methods commonly used at present are the CLS and CML. For the CLS method, the estimation of the threshold variable *r* can be achieved based on the following steps:

*Step 1*. Denote $\underline{r}$ and $\bar{r}$ as the 10th and 90th quantile value of the observations $\left\{X_{1},\ldots ,X_{n}\right\}$, for each $r\in [\underline{r},\bar{r}]\cap N$, and find ${\hat{r}}_{CLS}$ such that
  $$\begin{array}{c} {\hat{r}}_{CLS}=\arg\min_{r\in [\underline{r},\bar{r}]\cap N}Q\left(\theta (r)\right). \end{array}$$
*Step 2*. The parameter vector ${\hat{\theta }}_{CLS}\left({\hat{r}}_{CLS}\right)$ is estimated by (6) under the estimator ${\hat{r}}_{CLS}$, and all the parameters under *r* unknown cases are as follows:
  $$\begin{array}{c} {\hat{\eta }}_{CLS}={\left({\hat{\theta }}_{CLS}^{⊤}\left({\hat{r}}_{CLS}\right),{\hat{r}}_{CLS}\right)}^{⊤}. \end{array}$$
Similarly, the CML estimates for the threshold variable *r* can also be achieved based on the following steps:
*Step 1*. Denote $\underline{r}$ and $\bar{r}$ as the 10th and 90th quantile value of the observations $\left\{X_{1},\ldots ,X_{n}\right\}$, for each $r\in [\underline{r},\bar{r}]\cap N$, and find ${\hat{r}}_{CML}$ such that
  $$\begin{array}{c} {\hat{r}}_{CML}=\arg\max_{r\in [\underline{r},\bar{r}]\cap N}L\left(\theta (r)\right). \end{array}$$
*Step 2*. The parameter vector ${\hat{\theta }}_{CML}\left({\hat{r}}_{CML}\right)$ is estimated by (6) under the estimator ${\hat{r}}_{CML}$, and all the parameters under *r* unknown cases are as follows:
  $$\begin{array}{c} {\hat{\eta }}_{CML}={\left({\hat{\theta }}_{CML}^{⊤}\left({\hat{r}}_{CML}\right),{\hat{r}}_{CML}\right)}^{⊤}. \end{array}$$
Similar procedures can be found in [13,14].

### 3.4. Testing the Existence of the Piecewise Structure

Threshold models are typically characterized by piecewise linearization, which divides a complex system into regimes using a specific threshold. Therefore, testing to detect the existence of segmented structures is very necessary. To date, many researchers have come up with different test statistics. A common high-performance method is to construct the likelihood ratio (LR) test based on the conditional likelihood function; see [14]. However, the LR test cannot be implemented because the TVMTTINAR(1) model is constructed by two operators. In this paper, we constructed the Wald test statistics to detect the existence of piecewise structures in the TVMTTINAR(1) model. The null hypothesis and the alternative hypothesis take the form:

(10) $$\begin{array}{c} H_{0}^{\left(1\right)}:\;\beta_{1}=\beta_{2}\;\;\;\;\mathrm{vs}.\;\;\;\;H_{1}^{\left(1\right)}:\;\beta_{1}\neq \beta_{2}. \end{array}$$

Note that, although the TVMTTINAR(1) model does not degenerate to the INAR-type model when $\beta_{1}=\beta_{2}$, the probability of this happening is extremely small and will not be considered here. That is to say, we only prove the existence of the piecewise structure by testing $\beta_{1}\neq \beta_{2}$. A simple idea, learned from [22], is to use the test of the difference between two normal population means based on the asymptotic normality of some consistent estimators. Then, we construct the Wald test based on the asymptotic distribution (9) of the CLS estimator ${\hat{\theta }}_{CLS}$ and obtain the following result.

Let $\hat{\Sigma }={\hat{V}}^{-1}\hat{W}{\hat{V}}^{-1}$, where $\hat{V}$ and $\hat{W}$ are square matrices of order 2q+3 with the jkth element given by

$${\hat{V}}_{jk}:={\left(\frac{1}{n}\sum_{t=1}^{n}\frac{\partial }{\partial \theta_{j}}g\left(\theta ,X_{t-1},Z_{t}\right)\frac{\partial }{\partial \theta_{k}}g\left(\theta ,X_{t-1},Z_{t}\right)\right)}_{\theta ={\hat{\theta }}_{CLS}},$$

$${\hat{W}}_{jk}:={\left(\frac{1}{n}\sum_{t=1}^{n}U_{t}^{2}\left(\theta \right)\frac{\partial }{\partial \theta_{j}}g\left(\theta ,X_{t-1},Z_{t}\right)\frac{\partial }{\partial \theta_{k}}g\left(\theta ,X_{t-1},Z_{t}\right)\right)}_{\theta ={\hat{\theta }}_{CLS}}.$$

Obviously, they are the consistent estimators of V and W (defined in Theorem 1). Then, the statistic for testing the problem (10) is defined by

$$\begin{array}{c} T_{n}^{\left(1\right)}=\frac{I({\hat{\beta }}_{1,CLS}-{\hat{\beta }}_{2,CLS})}{\sqrt{A\hat{\Sigma }A^{⊤}/n}}, \end{array}$$

where $I={\left(1,\ldots ,1\right)}_{1\times (q+1)}$, $A={\left(I,-I\right)}_{1\times 2(q+1)}$. Furthermore, under $H_{0}^{\left(1\right)}$,

$$\begin{array}{c} T_{n}^{\left(1\right)}\overset{d}{⟶}N\left(0,1\right),\;\;\;\mathrm{as}\;\;\;n\to \infty . \end{array}$$

### 3.5. Testing the Existence of Explanatory Variables

The existence of observable explanatory variables in the TVMTTINAR(1) model constructs time-varying characteristics. Once the explanatory variables are not present, the model degrades to a constant-coefficient mixture thinning operator threshold INAR model (MTTINAR(1)), i.e.,

(11) $$X_{t}=\left(\alpha_{1}\circ X_{t-1}\right)I_{1,t}^{R}+\left(\alpha_{2}*X_{t-1}\right)I_{2,t}^{R}+\varepsilon_{t},\;\;t\in Z,$$

where $\alpha_{i}\in \left(0,1\right),i=1,2.$ Therandom variables are similar to Definition 1. A more-general version of such a problem is to test whether the explanatory variable coefficients $\beta_{i,j}\left(i=1,2,j=1,2,\ldots ,q\right)$ in each regime are all zeros, i.e.,

(12) $$\begin{array}{c} H_{0}^{\left(2\right)}:\;\beta_{i,j}=0,i=1,2,j=1,\ldots ,q\;\;\mathrm{vs}.\;\;H_{1}^{\left(2\right)}:\;\mathrm{At}\;\mathrm{least}\;\mathrm{one}\;\beta_{i,j}\neq 0,i\in \left\{1,2\right\},1\leq j\leq q. \end{array}$$

For this, we construct the following two test statistics. The first method is to construct a test statistic using the asymptotic normality of the estimator ${\hat{\theta }}_{CLS}$. Let $0_{j\times k}$ be a zero matrix with *j* rows and *k* columns, $B=(0_{q\times 1},I_{q\times q})$, $C=\left(\begin{array}{cc} B & 0_{q\times (q+1)}\\ 0_{q\times (q+1)} & B \end{array}\right)$. We construct the test statistic as follows:

$$\begin{array}{c} T_{n}^{\left(2\right)}=n{\hat{\theta }}_{CLS}^{⊤}C^{⊤}{\left(C\hat{\Sigma }C^{⊤}\right)}^{-1}C{\hat{\theta }}_{CLS}. \end{array}$$

Then, under $H_{0}^{\left(2\right)}$,

$$\begin{array}{c} T_{n}^{\left(2\right)}\overset{d}{⟶}\chi_{2q}^{2},\;\;\;\mathrm{as}\;\;\;n\to \infty . \end{array}$$

Another approach is to construct a classical likelihood ratio (LR) test statistic. Let $\tilde{\theta }:={\left(\alpha_{1},\alpha_{2},\lambda \right)}^{⊤}$ be the parameter of the MTTINAR(1) model with the parameter set $\Theta_{\tilde{\theta }}$:

$$\Theta_{\tilde{\theta }}=\left\{\tilde{\theta }\in \left(0,1\right)\times \left(0,1\right)\times \left(0,+\infty \right)\right\}.$$

Then, the LR statistic for testing problem (12) is defined by

$$\begin{array}{c} T_{n}^{\left(3\right)}=2(\max_{\Theta_{\theta }}L\left(\theta \right)-\max_{\Theta_{\tilde{\theta }}}\tilde{L}\left(\tilde{\theta }\right)), \end{array}$$

where $\tilde{L}\left(\tilde{\theta }\right)$ is the conditional log-likelihood function for the MTTINAR(1) model (11). Suppose we have a series of observations ${\left\{x_{t}\right\}}_{t=1}^{n}$ generated from the MTTINAR(1) process, then $\tilde{L}\left(\tilde{\theta }\right)$ is given by

L˜(θ˜):=∑t=1nℓ˜t(θ˜)=∑t=1nlogP˜(xt−1,xt),P˜(xt−1,xt)=I1,tR∑m=0min(xt−1,xt)xt−1me−λλxt−m(xt−m)!α1m(1−α1)xt−1−m+I2,tR∑m=0xtΓ(xt−1+m)Γ(xt−1)Γ(m+1)α2m(1+α2)xt−1+me−λλxt−m(xt−m)!.

Furthermore, under $H_{0}^{\left(2\right)}$,

$$\begin{array}{c} T_{n}^{\left(3\right)}\overset{d}{⟶}\chi_{2q}^{2},\;\;\;\mathrm{as}\;\;\;n\to \infty . \end{array}$$

## 4. Simulation Studies

To evaluate the finite-sample performance of the proposed inference methods and testing statistics, we conducted extensive simulation studies and split the simulation studies into the following four parts. In the first two parts, we considered the performance of the CLS and CML estimators in two cases where threshold *r* is known and unknown. In the third and forth parts, we mainly focused on the performance of the proposed test statistics by empirical sizes and powers.

To get started, we first introduce the following models applied to Section 4.1 and Section 4.2. The models are divided into the A-type and B-type models, which represent the R=0 and R=1 TVMTTINAR(1) models, respectively. The two types of models choose similar parameters. In order to save space, we introduce the A-type model, while the parentheses represent the similar B-type model:

**Model A1 (B1)**: Generated from the TVMTTINAR(1) process (3) with R=0 (R=1), λ=5, r=6, $\left(\beta_{1,0},\beta_{1,1}\right)=\left(0.1,0.3\right)$, $\left(\beta_{2,0},\beta_{2,1}\right)=\left(-0.5,-0.6\right)$. The explanatory variables $Z_{1,t}$ are generalized from the i.i.d. normal distribution N(0,1).
**Model A2 (B2)**: Generated from the TVMTTINAR(1) process (3) with R=0 (R=1), λ=5, r=6, $\left(\beta_{1,0},\beta_{1,1}\right)=\left(0.3,0.3\right)$, $\left(\beta_{2,0},\beta_{2,1}\right)=\left(-0.5,-0.6\right)$. The explanatory variables $Z_{1,t}$ are generalized from an AR(1) process, i.e., $Z_{1,t}=0.5Z_{1,t-1}+\epsilon_{t}$ with $Z_{1,0}=0$, $\epsilon_{t}∼N\left(0,1\right)$.
**Model A3 (B3)**: Generated from the TVMTTINAR(1) process (3) with R=0 (R=1), λ=5, r=6, $\left(\beta_{1,0},\beta_{1,1},\beta_{1,2}\right)=\left(0.1,0.5,0.3\right)$, $\left(\beta_{2,0},\beta_{2,1},\beta_{2,2}\right)=\left(-0.3,-0.5,-0.6\right)$. The explanatory variables $Z_{1,t}$ are generalized from an AR(1) process, i.e., $Z_{1,t}=0.5Z_{1,t-1}+\epsilon_{t}$ with $Z_{1,0}=0$, $\epsilon_{t}∼N\left(0,1\right)$; $Z_{2,t}$ is generalized from a seasonal series, i.e., $Z_{2,t}=\sin\left(2\pi t/12\right)+\epsilon_{t}$ with $\epsilon_{t}∼N\left(0,0.25\right)$.

All simulations were implemented in MATLAB. The sample size considered in all simulations was n=200,500,1000. For each model, the value of *r* was chosen such that the observations in each regime comprised at least 20% of the total sample size. The empirical results displayed in the tables and box plots, that is the empirical biases and mean square errors (MSEs), were computed over 10,000 replications.

### 4.1. Simulation Study When *r* Is Known

Table 1 and Table 2 report the bias and MSE of the CLS and CML estimators for Models A1–B3 when *r* is known. It is easy to see that all the simulation results performed better as *n* increased, which implies that the two estimation methods can lead to good and consistent estimators when *r* is known. It is worth mentioning that, although it is not mentioned in the main Conclusion, the simulation showed that the CML estimators are consistent. In addition, ${\hat{\theta }}_{CML}$ has smaller bias and MSE, which means that ${\hat{\theta }}_{CML}$ is better than ${\hat{\theta }}_{CLS}$.

**Figure 1:**
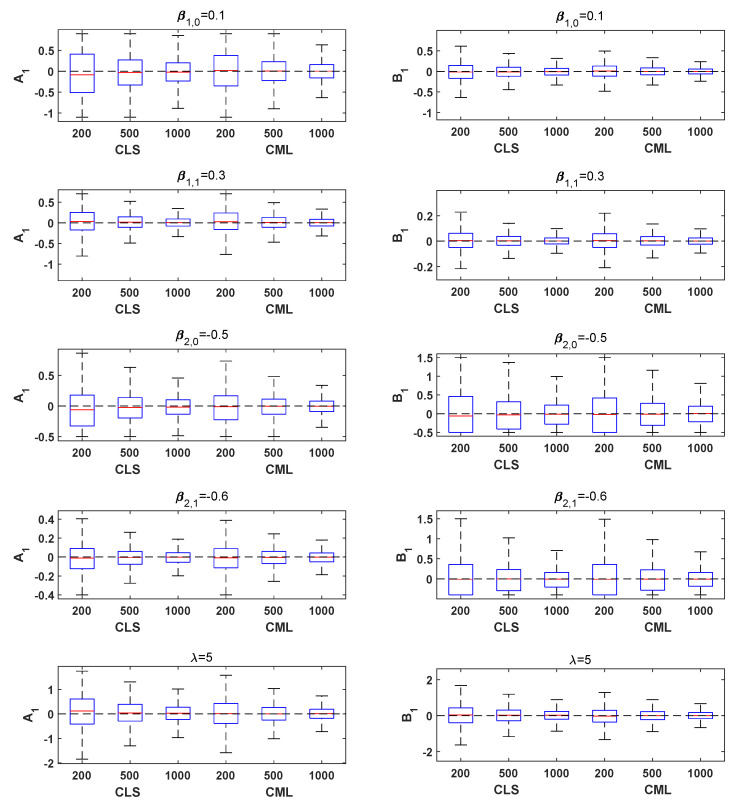
Box plots from 10,000 CLS and CML simulation estimators for Models A1 and B1, with the sample size n=200,500,1000.

**Figure 2:**
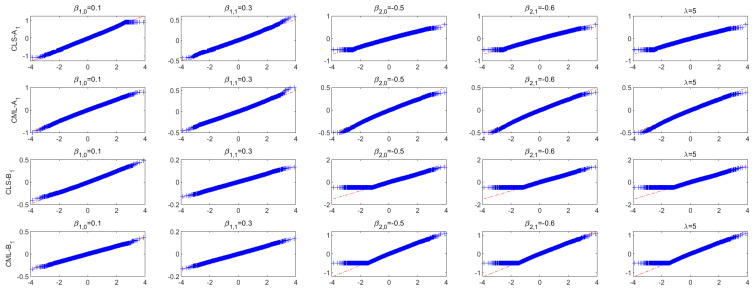
QQ plots of CLS and CML estimators for Models A1 and B1, with the sample size n=200.

For the sake of intuition, we also give the box plots and QQ plots of the CLS and CML estimators. Figure 1 plots the bias of 10,000 CLS and CML simulation estimators for Models A1 and B1. Note that the box plots are symmetric and centered on zero-bias; both the bias and MSE for the CML estimators are smaller than the CLS estimators, which is consistent with the previous conclusions. Figure 2 shows the QQ plots of the CLS and CML estimators for Models A1 and B1 with the sample size n=1000. It is easy to see that the CLS and CML estimators are asymptotically normal for all parameters, especially for the CML estimator without the asymptotically normal theorem. Similar results were obtained for the remaining models, and the figures are omitted here to save space.

### 4.2. Simulation Study When *r* Is Unknown

Table 3 and Table 4 report the performance of the proposed CLS and CML estimators in Section 3.3 for Models A1–B3 when *r* is known. It is easy to draw the following conclusions from the tabular results. For small sample sizes (such as n=200), the bias and MSE of the estimator are still relatively large. However, with the increase of the sample size, this deviation decreases very quickly, mainly because the accuracy of threshold estimation is greatly improved with the increase of the sample size. Moreover, the CML estimator demonstrates a noticeable advantage over the CLS estimator. Nevertheless, this does not imply that CLS estimators lack any advantages. Table 5 reports the percentage of correctly identifying *r* (Frequency) and average time (s) across 10,000 replications. Without the closed-form solutions of the two methods, the CLS estimation method is still very advantageous in the calculation speed.

### 4.3. Empirical Sizes and Powers of the Wald Test

Some simulations were conducted to investigate the performances of the Wald test $T_{n}^{\left(1\right)}$. We selected the significance level as α=0.05 (the associated critical value was 1.65). For analyzing the empirical size, we first introduced the following two time-varying integer-valued autoregressive models (TVINAR).

(13) $$\begin{array}{ccc} \; & \mathrm{TVINAR}(1)\text{-}B:\;\;\;X_{t}=\frac{\exp(Z_{t}^{⊤}\beta_{1})}{1+\exp(Z_{t}^{⊤}\beta_{1})}\circ X_{t-1}+\varepsilon_{t}, & \end{array}$$

(14) $$\begin{array}{ccc} \; & \mathrm{TVINAR}(1)\text{-}G:\;\;\;X_{t}=\frac{\exp(Z_{t}^{⊤}\beta_{2})}{1+\exp(Z_{t}^{⊤}\beta_{1})}*X_{t-1}+\varepsilon_{t}. & \end{array}$$

where the explanatory variables $Z_{1,t}$ are generalized from an AR(1) process, i.e., $Z_{1,t}=-0.5*Z_{1,t-1}+\epsilon_{t}$ with $Z_{1,0}=0$, $\epsilon_{t}∼N\left(0,1\right)$; $Z_{2,t}$ is generalized from a seasonal series, i.e., $Z_{2,t}=\sin\left(2\pi t/12\right)+\epsilon_{t}$ with $\epsilon_{t}∼N\left(0,0.25\right)$.

For analyzing the empirical size, Models $T_{11}$–$T_{22}$ were considered. For analyzing the empirical power, Models $T_{31}$–$T_{32}$ were considered:

**Model $T_{11}$**: Generated from the TVINAR(1)-B process (13) with $\left(\beta_{1}^{⊤},\lambda \right)$$=\left(\beta_{1,0},\beta_{1,1},\beta_{1,2},\lambda \right)=\left(0.1,0.5,0.3,5\right)$.
**Model $T_{12}$**: Generated from the TVINAR(1)-B process (13) with $\left(\beta_{1}^{⊤},\lambda \right)$$=\left(\beta_{1,0},\beta_{1,1},\beta_{1,2},\lambda \right)=\left(-0.7,-0.8,0.6,2\right)$.
**Model $T_{21}$**: Generated from the TVINAR(1)-G process (14) with $\left(\beta_{2}^{⊤},\lambda \right)$$=\left(\beta_{2,0},\beta_{2,1},\beta_{2,2},\lambda \right)=\left(-0.3,-0.3,-0.6,2\right)$.
**Model $T_{22}$**: Generated from the TVINAR(1)-G process (14) with $\left(\beta_{2}^{⊤},\lambda \right)$$=\left(\beta_{2,0},\beta_{2,1},\beta_{2,2},\lambda \right)=\left(-0.6,0.8,-0.5,7\right)$.
**Model $T_{31}$**: Generated from the TVMTTINAR(1) process (3) with R=0, λ=5, r=6, $\beta_{1}^{⊤}=\left(\beta_{1,0},\beta_{1,1},\beta_{1,2}\right)=\left(0.1,0.5,0.3\right)$, $\beta_{2}^{⊤}=\left(\beta_{2,0},\beta_{2,1},\beta_{2,2}\right)=\left(-0.3,-0.3,-0.6\right)$. The explanatory variables $Z_{1,t}$ are generalized from an AR(1) process, i.e., $Z_{1,t}=-0.5*Z_{1,t-1}+\epsilon_{t}$ with $Z_{1,0}=0$, $\epsilon_{t}∼N\left(0,1\right)$; $Z_{2,t}$ is generalized from a seasonal series, i.e., $Z_{2,t}=\sin\left(2\pi t/12\right)+\epsilon_{t}$ with $\epsilon_{t}∼N\left(0,0.25\right)$.
**Model $T_{32}$**: Generated from the TVMTTINAR(1) process (3) with R=1, λ=3, r=6, $\beta_{1}^{⊤}=\left(\beta_{1,0},\beta_{1,1},\beta_{1,2}\right)=\left(-0.4,-0.8,0.3\right)$, $\beta_{2}^{⊤}=\left(\beta_{1,0},\beta_{1,1},\beta_{1,2}\right)=\left(-0.3,0.7,-0.4\right)$. The explanatory variables $Z_{1,t}$ are generalized from an AR(1) process, i.e., $Z_{1,t}=-0.5*Z_{1,t-1}+\epsilon_{t}$ with $Z_{1,0}=0$, $\epsilon_{t}∼N\left(0,1\right)$; $Z_{2,t}$ is generalized from a seasonal series, i.e., $Z_{2,t}=\sin\left(2\pi t/12\right)+\epsilon_{t}$ with $\epsilon_{t}∼N\left(0,0.25\right)$.

The results are reported in Table 6. As can be seen from Table 6, for the empirical sizes, the Wald test $T_{n}^{\left(1\right)}$ gave satisfactory performances and the empirical sizes for Models $T_{11}$–$T_{22}$ were closer to the significant level of α=0.05 as the sample size increased. For the empirical powers, Table 6 also indicates that the $T_{n}^{\left(1\right)}$ succeeded in showing high values in almost each case. The above discussion shows the success of the proposed Wald test $T_{n}^{\left(1\right)}$ to detect the existence of the piecewise structure.

### 4.4. Empirical Sizes and Powers of the Proposed Test in Section 3.5

Similarly, we investigated the performances of the test $T_{n}^{\left(2\right)}$ and the LR test $T_{n}^{\left(3\right)}$ with the following models:

**Model $T_{41}$**: Generated from the MTTINAR(1) process (11) with $\left(\alpha_{1},\alpha_{2},\lambda ,r,R\right)$ = (0.6225,0.4502,5,6,0).
**Model $T_{42}$**: Generated from the MTTINAR(1) process (11) with $\left(\alpha_{1},\alpha_{2},\lambda ,r,R\right)$ = (0.4502,0.6225,3,4,1).
**Model $T_{51}$**: Generated from the TVMTTINAR(1) process (3) with R=0, λ=5, r=6, $\beta_{1}^{⊤}=\left(\beta_{1,0},\beta_{1,1}\right)=\left(0.5,0.3\right)$, $\beta_{2}^{⊤}=\left(\beta_{2,0},\beta_{2,1}\right)=\left(-0.2,-0.2\right)$. The explanatory variables $Z_{1,t}$ are generalized from the i.i.d. uniform distribution U(−10,1).
**Model $T_{52}$**: Generated from the TVMTTINAR(1) process (3) with R=1, λ=5, r=6, $\beta_{1}^{⊤}=\left(\beta_{1,0},\beta_{1,1}\right)=\left(0.5,0.3\right)$, $\left(\beta_{2}^{⊤}\right)=\left(\beta_{2,0},\beta_{2,1}\right)=\left(-0.2,-0.2\right)$. The explanatory variables $Z_{1,t}$ are generalized from the i.i.d. uniform distribution U(−10,1).

Models $T_{41}$ and $T_{42}$ were used to analyze the empirical sizes; Models $T_{41}$ and $T_{42}$ were applied to analyze the empirical powers. We selected the significance level of α=0.05 (since q=1, the associated critical value was 5.991). Table 7 shows the empirical sizes and powers result. It is easy to see that, for the empirical sizes, both tests gave satisfactory performances and the empirical sizes for Models $T_{41}$–$T_{42}$ were closer to the significant level of α=0.05 as the sample size increased. For the empirical power, both proposed tests were increasingly close to 1 as the sample size increased in each case. In addition, although both tests can successfully detect the existence of the explanatory variables, the LR test performed significantly better.

## 5. Real Data Example

In this section, we will utilize the TVMTTINAR(1) model to match the daily stock trading volume dataset of an automotive company, Volkswagen Corporation (VOW). There are some explanations for the selection of the factors affecting the trading volume of stocks in the automotive industry. The volume of stock trading in the automotive industry can be affected by a number of factors, the most well-known of which are the state of the economy and the state of the oil market. After all, the state of the economy largely determines consumers’ ability and willingness to buy. On the one hand, the fluctuation of oil prices increases the production cost, and on the other hand, it affects the willingness of consumers to buy traditional fuel vehicles and directly or indirectly affects the automobile industry.

Economic conditions can be represented by some stock market indices. We selected the Dow Jones Industrial average indices (DJI) here. The DJI can reflect the overall performance of the stock market and is also used as an indicator of the health of the economy. Therefore, it is reasonable to choose the DJI’s stock data series as economic indicators. On the other hand, oil prices vary widely between countries and regions, making it difficult to find a uniform measure, the Crude oil (Co) stock data series was selected here to represent the oil market.

All datasets were originally downloaded online from the Yahoo finance web site (https://hk.finance.yahoo.com/, accessed on 7 December 2010). Both the DJI and Co stock data series include open, high, low, close, and adjusted (Adj) close prices, wherein the Adj close price datasets were selected as the explanatory variables for the analysis. In addition, the two explanatory variable datasets need to be differentiated to reflect fluctuations in the economy and the oil market.

### 5.1. Volkswagen Corporation Daily Stock Trading Volume Data

**Figure 3:**
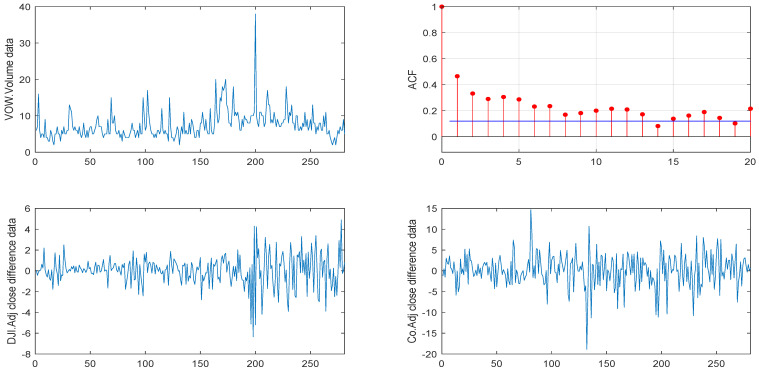
Sample path and ACF of the VOW daily stock trading volume dataset (from 7 December 2010 and ending in 11 January 2012).

We first considered the VOW daily stock trading volume dataset, which consist of 281 observations starting in 7 December 2010 and ending in 11 January 2012. As the data are relatively large, considering the convenience of calculation, we analyzed the data by the unit of a “2 × $10^{5}$” trading volume. Figure 3 shows the sample path and the sample autocorrelation (ACF) of the observations, where the first line shows the sample path and ACF of the VOW daily stock trading volume dataset and the second line shows the sample path after the difference in the DJI and Co’s Adj close prices.

Next, we used the TVMTTINAR(1) model and the following integer-valued threshold autoregressive models to fit the VOW corporation dataset and compare different models via the Akaike Information Criterion (AIC) and Bayesian Information Criterion (BIC):

- SETINAR(2,1) model [12].
- NBTINAR(1) model [15].
- RCTINAR(1) model [11].
- BiNB-MTTINAR(1) (R=0) model [17].
- BiNB-MTTINAR(1) (R=1) model [17].

For each of the above models, we estimated the CML of the parameters and the threshold *r* with the range r∈{4,5,…,12}, where 4 and 12 are the 10th and 90th quantiles of the data. Furthermore, the standard error (SE) of ${\hat{\theta }}_{CML}$, the root mean square of the differences between the observations and forecasts (RMS), and the AIC and BIC values are also given. Among them, the standard error for the CML estimator can be obtained as the square roots of the elements in the diagonal of the inverse of the negative Hessian of the log-likelihood calculated at the CML estimates. The RMS is defined as follows:

$$\mathrm{RMS}=\sqrt{\frac{1}{n-1}\sum_{t=2}^{n}{\left(X_{t}-\sum_{i=1}^{2}\frac{\exp(Z_{t}^{⊤}\beta_{i})}{1+\exp(Z_{t}^{⊤}\beta_{i})}X_{t-1}I_{i,t}^{R}-\lambda \right)}^{2}}.$$

The fitting results are summarized in Table 8. As seen from the results presented in Table 8, the proposed TVMTTINAR(1) (R=0) model outperformed the other SETINAR models when considering the AIC as an information criterion. However, due to the excessive number of parameters, when considering the BIC as an information criterion, the model appeared slightly less favorable. Additionally, the TVMTTINAR(1) (R=0) model had the lowest RMS value. Taking all factors into account, the TVMTTINAR(1) (R=0) model was highly competitive, making it reasonable to apply it for fitting this VOW dataset.

Then, we computed the (standardized) Pearson residuals ${\mathrm{Pr}}_{t}(\hat{\theta }$) to check if the fit model was adequate for the data.

(15) $$\begin{array}{c} {\mathrm{Pr}}_{t}\left(\hat{\theta }\right)=\frac{X_{t}-\sum_{i=1}^{2}\frac{\exp(Z_{t}^{⊤}\beta_{i})}{1+\exp(Z_{t}^{⊤}\beta_{i})}X_{t-1}I_{i,t}^{R}-\lambda }{\sqrt{\frac{\exp(Z_{t}^{⊤}\beta_{1})}{{\left[1+\exp\left(Z_{t}^{⊤}\beta_{1}\right)\right]}^{2}}X_{t-1}I_{1,t}^{R}+\frac{\exp\left(Z_{t}^{⊤}\beta_{2}\right)\left[1+2\exp\left(Z_{t}^{⊤}\beta_{2}\right)\right]}{{\left[1+\exp\left(Z_{t}^{⊤}\beta_{2}\right)\right]}^{2}}X_{t-1}I_{1,t}^{R}+\lambda ,}}. \end{array}$$

We proceeded to apply the TVMTTINAR(1) (R=0) model to fit this dataset and computed some additional fitting-related information beyond Table 8. These details are summarized in Table 9, encompassing the proportion of samples below the threshold value relative to the total sample size (rate), the test statistic $T_{n}^{\left(1\right)}$ for testing the presence of the segmented structure, the test statistic $T_{n}^{\left(3\right)}$ for testing the existence of explanatory variables, along with the mean and variance of the Pearson residuals.

After computing, the proportion of estimated values below the threshold was 0.5018, indicating reliable results on both sides of the threshold. $T_{n}^{\left(1\right)}=2.2343$, surpassing the critical value of 1.65 at a 0.05 significance level, leading us to reject the null hypothesis $H_{0}^{\left(1\right)}:\beta_{1}=\beta_{2}$, confirming the presence of a segmented structure. $T_{n}^{\left(3\right)}=35.2307$, exceeding the critical value of 9.487 at a 0.05 significance level, compelling us to accept the alternative hypothesis $H_{1}^{\left(2\right)}:\;\mathrm{At}\;\mathrm{least}\;\mathrm{one}\;\beta_{i,j}\neq 0,i\in \left\{1,2\right\},1\leq j\leq q.$ Although the TVMTTINAR(1) (R=0) model in Table 8 demonstrates superiority in terms of the AIC and RMS, its superiority is not immediately evident. However, it is important to highlight that these test results fully justify the introduction of mixture thinning operators and observable explanatory variables. This provides another level of evidence supporting the suitability of the TVMTTINAR(1) (R=0) model for fitting this dataset, thus highlighting the model’s competitiveness. Additionally, the model’s fit Pearson residuals exhibited a mean of 0.0041 and a variance of 1.1116, indicating a well-balanced fit.

**Figure 4:**
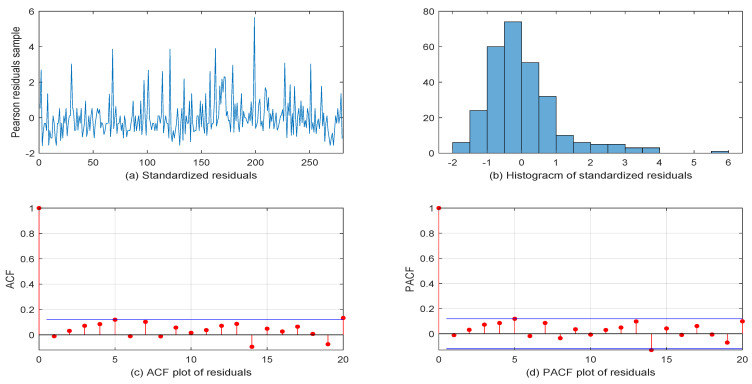
Diagnostic checking plots for the first VOW dataset.

Finally, Figure 4 shows the diagnostic checking plots for our out fit model, including the (a) standardized residuals, (b) Histogram of the standardized residuals, (c) ACF plot of the residuals, and (d) PACF plot of the residuals. From Figure 4, it can be seen that the Pearson residual samples’ ACF and PACF had values close to zero, which reveals that our fit model was more suitable.

### 5.2. Another VOW Daily Stock Trading Volume Dataset

**Figure 5:**
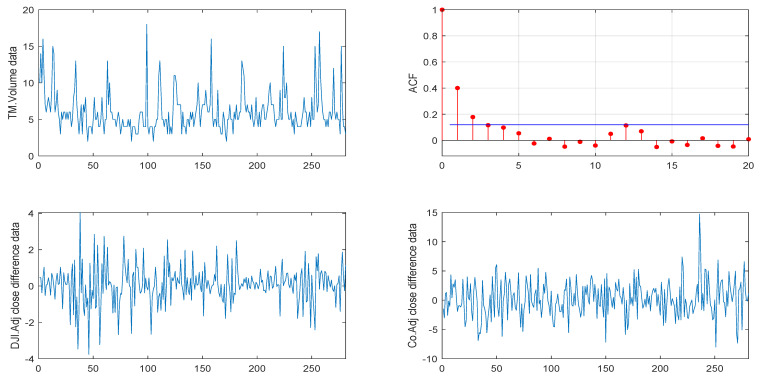
Sample path and ACF of the VOW daily stock trading volume dataset (from 7 December 2010 and ending in 6 June 2011).

Similar to the first data analysis, we considered another dataset of the VOW daily stock trading volume, which also consists of 281 observations starting in 4 May 2010 and ending in 6 June 2011. As the data are relatively large, considering the convenience of calculation, we analyzed the data by the unit of a “2 $\times \;10^{5}$” trading volume. Also, Figure 5 shows the sample path and the sample autocorrelation (ACF) of the observations.

Next, we compared the performances of SETINAR(2,1), NBTINAR(1), RCTINAR(1), and BiNB-MTTINAR(1) versus TVMTTINAR(1) for this series. The estimation results are shown in Table 10. Clearly, for this dataset, the TVMTTINAR(1) (R=1) model demonstrated superior performance in terms of the AIC and RMS. Additionally, it is worth noting that, while both sets of data analysis belong to the VOW dataset, particularly with overlapping data (from 7 December 2010 to 6 June 2011), the optimal model selection changed. This indicates the presence of a change point in this period, suggesting a need for further discussion and analysis in subsequent research.

Then, we also summarize the rate, the test statistic $T_{n}^{\left(1\right)}$, $T_{n}^{\left(3\right)}$, and the mean and variance of the Pearson residuals in Table 11. After computing, the proportion of estimated values below the threshold was 0.5267, indicating reliable results on both sides of the threshold. $T_{n}^{\left(1\right)}=1.6682$, surpassing the critical value of 1.65 at a 0.05 significance level, leading us to reject the null hypothesis $H_{0}^{\left(1\right)}:\beta_{1}=\beta_{2}$, confirming the presence of a segmented structure. $T_{n}^{\left(3\right)}=63.9769$, exceeding the critical value of 9.487 at a 0.05 significance level, compelling us to accept the alternative hypothesis $H_{1}^{\left(2\right)}:\;\mathrm{At}\;\mathrm{least}\;\mathrm{one}\;\beta_{i,j}\neq 0,i\in \left\{1,2\right\},1\leq j\leq q.$ Also, it is worth noting that the TVMTTINAR(1) (R=1) model in Table 10 exhibited some superiority in terms of the AIC and RMS. However, the degree of superiority was not clearly evident. It is important to mention that the aforementioned test results thoroughly justified the need to introduce mixture thinning operators and observable explanatory variables. These findings provide further evidence that the TVMTTINAR(1) (R=1) model is highly suitable for fitting the dataset, thereby highlighting its competitive nature. Additionally, the model’s fit Pearson residuals exhibited a mean of 0.0015 and a variance of 1.0148, indicating a well-balanced fit.

**Figure 6:**
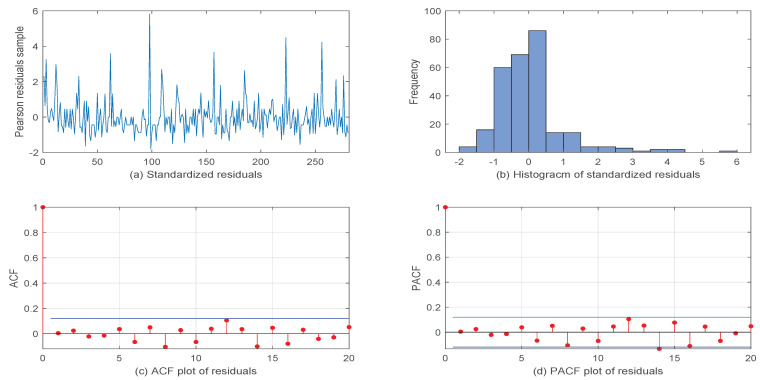
Diagnostic checking plots for another VOW dataset.

Finally, Figure 6 shows the diagnostic checking plots for our out fit model, including the (a) standardized residuals, (b) Histogram of the standardized residuals, (c) ACF plot of the residuals, and (d) PACF plot of the residuals. From Figure 4, it can be seen that the Pearson residual samples’ ACF and PACF had values close to zero, which reveals that our fitted model was suitable.

## 6. Conclusions

This article introduces a first-order time-varying coefficient mixture thinning threshold integer-valued autoregressive process. The process was proven to be stationary and ergodic. We investigated the CLS and CML techniques for parameter estimation, and the asymptotic properties of the estimators were demonstrated. Two methods were suggested for estimating the unknown threshold parameter *r*, based on the CLS and CML score functions. Additionally, we constructed the Wald test statistic to check for the existence of the piecewise structure and constructed two test statistics to test the existence of the explanatory variables. Finally, we successfully applied the TVMTINAR(1) model to Volkswagen Corporation’s daily stock trading volume datasets. From real data studies, potential problems for future research include extending the results to a mixture thinning threshold INAR model with random coefficients and studying a TVMTTINAR(1) model with change points. These will remain the subject of future research.

## Figures and Tables

**Table 1:** Simulation results for Models A1–B2 when *r* is known.

		A1				A2			
		CLS		CML		CLS		CML	
Sample Size	Para.	Bias	MSE	Bias	MSE	Bias	MSE	Bias	MSE
n=200	$\beta_{1,0}$	−0.0544	0.3579	0.0076	0.2669	−0.0856	0.3338	0.0071	0.2476
	$\beta_{1,1}$	0.0430	0.1017	0.0398	0.0955	0.0261	0.0915	0.0273	0.0882
	$\beta_{2,0}$	−0.0607	0.0991	−0.0302	0.0731	−0.0837	0.0931	−0.0419	0.0671
	$\beta_{2,1}$	−0.0216	0.0255	−0.0164	0.0229	−0.0186	0.0216	−0.0160	0.0189
	λ	0.0856	0.4181	0.0245	0.3047	0.1343	0.3908	0.0438	0.2752
n=500	$\beta_{1,0}$	−0.0245	0.1947	0.0020	0.1150	−0.0472	0.1886	0.0032	0.1149
	$\beta_{1,1}$	0.0209	0.0374	0.0156	0.0337	0.0158	0.0323	0.0141	0.0297
	$\beta_{2,0}$	−0.0324	0.0547	−0.0156	0.0344	−0.0464	0.0515	−0.0194	0.0313
	$\beta_{2,1}$	−0.0114	0.0105	−0.0077	0.0090	−0.0098	0.0085	−0.0077	0.0068
	λ	0.0449	0.2357	0.0141	0.1417	0.0778	0.2224	0.0214	0.1307
n=1000	$\beta_{1,0}$	−0.0157	0.1056	−0.0014	0.0564	−0.0190	0.1021	0.0046	0.0534
	$\beta_{1,1}$	0.0094	0.0169	0.0067	0.0154	0.0084	0.0152	0.0063	0.0135
	$\beta_{2,0}$	−0.0197	0.0300	−0.0101	0.0169	−0.0226	0.0283	−0.0075	0.0154
	$\beta_{2,1}$	−0.0070	0.0053	−0.0048	0.0045	−0.0058	0.0044	−0.0037	0.0035
	λ	0.0279	0.1324	0.0108	0.0717	0.0369	0.1239	0.0073	0.0645
		B1				B2			
		CLS		CML		CLS		CML	
Sample Size	Para.	Bias	MSE	Bias	MSE	Bias	MSE	Bias	MSE
n=200	$\beta_{1,0}$	−0.0084	0.0553	0.0089	0.0338	−0.0304	0.0466	0.0031	0.0271
	$\beta_{1,1}$	0.0057	0.0070	0.0045	0.0066	0.0052	0.0039	0.0041	0.0036
	$\beta_{2,0}$	0.0718	0.3459	0.0684	0.2918	0.1148	0.4075	0.1362	0.3766
	$\beta_{2,1}$	0.0583	0.2060	0.0587	0.2037	0.0767	0.2385	0.0745	0.2307
	λ	0.0100	0.3801	−0.0315	0.2366	0.0794	0.3770	−0.0158	0.2228
n=500	$\beta_{1,0}$	−0.0031	0.0257	0.0013	0.0147	−0.0135	0.0225	−0.0006	0.0121
	$\beta_{1,1}$	0.0024	0.0026	0.0018	0.0025	0.0026	0.0018	0.0019	0.0017
	$\beta_{2,0}$	0.0155	0.1914	0.0101	0.1454	0.0323	0.2214	0.0393	0.1878
	$\beta_{2,1}$	−0.0002	0.0982	0.0005	0.0951	0.0209	0.1220	0.0198	0.1179
	λ	0.0050	0.1838	−0.0043	0.1083	0.0370	0.1855	0.0003	0.1014
n=1000	$\beta_{1,0}$	−0.0053	0.0140	−0.0007	0.0077	−0.0100	0.0110	−0.0032	0.0058
	$\beta_{1,1}$	0.0012	0.0013	0.0007	0.0012	0.0014	0.0008	0.0010	0.0008
	$\beta_{2,0}$	−0.0084	0.1157	−0.0018	0.0860	−0.0045	0.1376	0.0055	0.1119
	$\beta_{2,1}$	−0.0163	0.0590	−0.0135	0.0567	−0.0127	0.0662	−0.0114	0.0639
	λ	0.0121	0.1020	0.0007	0.0578	0.0278	0.0934	0.0083	0.0504

**Table 2:** Simulation results for Models A3–B3 when *r* is known.

		A3				B3			
		CLS		CML		CLS		CML	
Sample Size	Para.	Bias	MSE	Bias	MSE	Bias	MSE	Bias	MSE
n=200	$\beta_{1,0}$	−0.0905	0.4153	0.0203	0.3111	−0.0285	0.0593	0.0005	0.0371
	$\beta_{1,1}$	0.0539	0.1056	0.0588	0.1019	0.0088	0.0070	0.0075	0.0065
	$\beta_{1,2}$	−0.0004	0.2013	0.0026	0.1929	0.0071	0.0126	0.0041	0.0118
	$\beta_{2,0}$	−0.0837	0.1029	−0.0360	0.0698	0.0269	0.3911	0.0432	0.3349
	$\beta_{2,1}$	−0.0179	0.0196	−0.0150	0.0172	−0.0159	0.1750	−0.0101	0.1707
	$\beta_{2,2}$	−0.0283	0.0371	−0.0203	0.0329	0.1030	0.2927	0.1025	0.2824
	λ	0.1419	0.4508	0.0369	0.3043	0.0598	0.3590	−0.0090	0.2323
n=500	$\beta_{1,0}$	−0.0467	0.2223	0.0027	0.1373	−0.0137	0.0258	−0.0014	0.0149
	$\beta_{1,1}$	0.0394	0.0423	0.0351	0.0392	0.0043	0.0025	0.0040	0.0023
	$\beta_{1,2}$	0.0183	0.0768	0.0148	0.0708	0.0040	0.0052	0.0028	0.0050
	$\beta_{2,0}$	−0.0398	0.0490	−0.0166	0.0300	−0.0221	0.2194	−0.0052	0.1678
	$\beta_{2,1}$	−0.0082	0.0069	−0.0066	0.0059	−0.0422	0.0848	−0.0352	0.0803
	$\beta_{2,2}$	−0.0118	0.0142	−0.0068	0.0121	0.0182	0.1339	0.0204	0.1276
	λ	0.0716	0.2330	0.0216	0.1395	0.0312	0.1635	0.0011	0.0974
n=1000	$\beta_{1,0}$	−0.0152	0.1161	0.0076	0.0638	−0.0089	0.0140	−0.0018	0.0077
	$\beta_{1,1}$	0.0222	0.0216	0.0172	0.0193	0.0016	0.0013	0.0013	0.0012
	$\beta_{1,2}$	0.0101	0.0356	0.0070	0.0329	0.0019	0.0025	0.0013	0.0023
	$\beta_{2,0}$	−0.0174	0.0252	−0.0053	0.0144	−0.0197	0.1215	−0.0047	0.0866
	$\beta_{2,1}$	−0.0037	0.0036	−0.0028	0.0032	−0.0282	0.0507	−0.0225	0.0470
	$\beta_{2,2}$	−0.0062	0.0071	−0.0028	0.0061	−0.0064	0.0781	−0.0025	0.0748
	λ	0.0305	0.1260	0.0049	0.0684	0.0202	0.0922	0.0025	0.0517

**Table 3:** Simulation results for Models A1–B2 when *r* is unknown.

		A1				A2			
		CLS		CML		CLS		CML	
Sample Size	Para.	Bias	MSE	Bias	MSE	Bias	MSE	Bias	MSE
n=200	$\beta_{1,0}$	−0.1673	0.4649	−0.0095	0.3159	−0.2118	0.4925	−0.0109	0.2932
	$\beta_{1,1}$	−0.0600	0.2097	0.0236	0.1342	−0.0609	0.1886	0.0198	0.1131
	$\beta_{2,0}$	−0.0811	0.0973	−0.0319	0.0738	−0.1050	0.0948	−0.0450	0.0680
	$\beta_{2,1}$	−0.0372	0.0451	−0.0259	0.0283	−0.0250	0.0350	−0.0222	0.0212
	λ	0.1526	0.4610	0.0338	0.3177	0.2131	0.4566	0.0547	0.2883
	*r*	1.0142	5.3096	0.2577	1.2313	0.8968	5.2976	0.1642	0.8174
n=500	$\beta_{1,0}$	−0.0566	0.2308	−0.0009	0.1222	−0.0682	0.2172	0.0014	0.1180
	$\beta_{1,1}$	0.0054	0.0536	0.0157	0.0367	0.0061	0.0416	0.0140	0.0307
	$\beta_{2,0}$	−0.0408	0.0566	−0.0166	0.0350	−0.0517	0.0534	−0.0199	0.0316
	$\beta_{2,1}$	−0.0187	0.0132	−0.0093	0.0093	−0.0123	0.0096	−0.0082	0.0069
	λ	0.0703	0.2566	0.0166	0.1451	0.0935	0.2394	0.0226	0.1321
	*r*	0.1719	0.8427	0.0181	0.0705	0.1054	0.6042	0.0067	0.0281
*n* = 1000	$\beta_{1,0}$	−0.0188	0.1093	−0.0015	0.0565	−0.0191	0.1023	0.0046	0.0534
	$\beta_{1,1}$	0.0080	0.0181	0.0067	0.0155	0.0083	0.0152	0.0064	0.0135
	$\beta_{2,0}$	−0.0207	0.0304	−0.0101	0.0169	−0.0226	0.0284	−0.0075	0.0154
	$\beta_{2,1}$	−0.0078	0.0055	−0.0048	0.0045	−0.0058	0.0044	−0.0037	0.0035
	λ	0.0304	0.1350	0.0109	0.0718	0.0370	0.1240	0.0073	0.0645
	*r*	0.0121	0.0411	0.0009	0.0059	0.0003	0.0003	−0.0001	0.0001
		B1				B2			
		CLS		CML		CLS		CML	
Sample Size	Para.	Bias	MSE	Bias	MSE	Bias	MSE	Bias	MSE
n=200	$\beta_{1,0}$	0.0394	0.0738	0.0146	0.0382	0.0001	0.0673	−0.0008	0.0333
	$\beta_{1,1}$	0.0164	0.0232	0.0078	0.0074	0.0200	0.0155	0.0066	0.0040
	$\beta_{2,0}$	0.3713	0.6543	0.1386	0.3408	0.5624	0.8281	0.3572	0.4869
	$\beta_{2,1}$	0.3190	0.4057	0.1768	0.2646	0.5179	0.5136	0.3818	0.3591
	λ	−0.2125	0.7856	−0.0367	0.3023	−0.1115	0.8718	0.0031	0.3169
	*r*	2.5127	15.9965	0.4938	1.2152	3.2435	25.3375	0.1035	1.0955
n=500	$\beta_{1,0}$	0.0188	0.0330	0.0021	0.0152	0.0361	0.0372	−0.0037	0.0137
	$\beta_{1,1}$	0.0077	0.0048	0.0025	0.0025	0.0114	0.0055	0.0025	0.0017
	$\beta_{2,0}$	0.1333	0.3180	0.0325	0.1566	0.5518	0.6754	0.3191	0.2900
	$\beta_{2,1}$	0.0926	0.1770	0.0390	0.1133	0.4852	0.4148	0.3708	0.2623
	λ	−0.0858	0.3009	−0.0053	0.1144	−0.1831	0.4647	0.0112	0.1245
	*r*	0.8616	5.2522	0.1170	0.1640	2.3496	18.1162	−0.1513	0.3999
n=1000	$\beta_{1,0}$	−0.0019	0.0150	−0.0007	0.0077	0.0423	0.0197	−0.0004	0.0063
	$\beta_{1,1}$	0.0020	0.0015	0.0008	0.0012	0.0072	0.0018	0.0013	0.0008
	$\beta_{2,0}$	0.0105	0.1346	0.0023	0.0884	0.5346	0.5129	0.3481	0.2311
	$\beta_{2,1}$	0.0005	0.0723	−0.0054	0.0608	0.4726	0.3270	0.3973	0.2285
	λ	−0.0011	0.1162	0.0008	0.0582	−0.1783	0.2391	−0.0017	0.0570
	*r*	0.1303	0.7299	0.0207	0.0231	1.2562	9.8408	−0.1482	0.2074

**Table 4:** Simulation results for Models A3–B3 when *r* is unknown.

		A3				B3			
		CLS		CML		CLS		CML	
Sample Size	Para.	Bias	MSE	Bias	MSE	Bias	MSE	Bias	MSE
n=200	$\beta_{1,0}$	−0.2088	0.4902	0.0025	0.3416	−0.0076	0.0699	0.0050	0.0394
	$\beta_{1,1}$	−0.1031	0.2460	0.0372	0.1274	0.0179	0.0152	0.0107	0.0069
	$\beta_{1,2}$	−0.1352	0.3164	−0.0169	0.2225	0.0144	0.0274	0.0055	0.0123
	$\beta_{2,0}$	−0.1069	0.1053	−0.0389	0.0711	0.1478	0.4684	0.0648	0.3516
	$\beta_{2,1}$	−0.0330	0.0369	−0.0219	0.0196	0.1450	0.3211	0.0455	0.2047
	$\beta_{2,2}$	−0.0315	0.0598	−0.0251	0.0365	0.2349	0.3862	0.1481	0.3138
	λ	0.2373	0.5380	0.0512	0.3236	−0.0311	0.5100	−0.0161	0.2623
	*r*	1.2538	7.7742	0.1827	0.8443	1.3943	9.2127	0.2116	0.5360
n=500	$\beta_{1,0}$	−0.0612	0.2341	0.0012	0.1395	−0.0109	0.0266	−0.0009	0.0150
	$\beta_{1,1}$	0.0284	0.0513	0.0352	0.0402	0.0052	0.0027	0.0042	0.0023
	$\beta_{1,2}$	0.0084	0.0855	0.0148	0.0721	0.0045	0.0057	0.0029	0.0050
	$\beta_{2,0}$	−0.0433	0.0499	−0.0171	0.0302	−0.0082	0.2310	−0.0029	0.1692
	$\beta_{2,1}$	−0.0106	0.0078	−0.0069	0.0059	−0.0301	0.0983	−0.0304	0.0834
	$\beta_{2,2}$	−0.0137	0.0151	−0.0071	0.0122	0.0305	0.1447	0.0251	0.1307
	λ	0.0837	0.2443	0.0228	0.1406	0.0206	0.1745	0.0001	0.0982
	*r*	0.0853	0.4439	0.0044	0.0140	0.1002	0.5810	0.0163	0.0277
n=1000	$\beta_{1,0}$	−0.0161	0.1170	0.0075	0.0639	−0.0088	0.0141	−0.0018	0.0077
	$\beta_{1,1}$	0.0216	0.0218	0.0172	0.0193	0.0017	0.0013	0.0013	0.0012
	$\beta_{1,2}$	0.0095	0.0359	0.0070	0.0330	0.0020	0.0025	0.0013	0.0023
	$\beta_{2,0}$	−0.0177	0.0253	−0.0053	0.0144	−0.0190	0.1224	−0.0045	0.0867
	$\beta_{2,1}$	−0.0039	0.0036	−0.0028	0.0032	−0.0280	0.0513	−0.0221	0.0473
	$\beta_{2,2}$	−0.0063	0.0071	−0.0028	0.0061	−0.0060	0.0785	−0.0020	0.0750
	λ	0.0313	0.1269	0.0049	0.0685	0.0198	0.0927	0.0024	0.0517
	*r*	0.0031	0.0103	0.0000	0.0006	0.0036	0.0228	0.0014	0.0020

**Table 5:** The performances of $\hat{r}$ for Models A1–B3.

		CLS		CML	
Model	Sample Size	Frequency	Average Time (s)	Frequency	Average Time (s)
A1	200	0.6330	0.5269	0.7841	2.3501
	500	0.9159	0.6343	0.9694	4.8531
	1000	0.9913	0.7666	0.9989	9.0141
A2	200	0.7164	0.4614	0.8564	2.2148
	500	0.9619	0.6495	0.9885	5.2616
	1000	0.9997	0.7582	0.9999	9.3752
A3	200	0.6700	0.6728	0.8479	3.8789
	500	0.9619	0.9298	0.9880	9.2877
	1000	0.9974	1.0712	0.9994	16.3961
		CLS		CML	
Model	Sample Size	Frequency	Average Time (s)	Frequency	Average Time (s)
B1	200	0.4341	0.3850	0.6511	2.0574
	500	0.7767	0.4581	0.8909	4.6666
	1000	0.9574	0.5514	0.9793	9.2278
B2	200	0.1633	0.4005	0.4463	2.4881
	500	0.2720	0.5062	0.6398	5.6456
	1000	0.5250	0.6105	0.7944	11.5242
B3	200	0.6513	0.4865	0.8069	3.1971
	500	0.9540	0.6448	0.9776	7.5834
	1000	0.9965	0.8010	0.9980	14.9456

**Table 6:** Empirical sizes and powers of $T_{n}^{\left(1\right)}$ at level 0.05.

Empirical Sizes, Significance Level α=0.05									
		Sample Size					Sample Size		
Model	Method	n=200	n=500	n=1000	Model	Method	n=200	n=500	n=1000
$T_{11}$	$T_{n}^{\left(1\right)}$	0.0497	0.0515	0.0517	$T_{12}$	$T_{n}^{\left(1\right)}$	0.0255	0.0517	0.0559
$T_{21}$	$T_{n}^{\left(1\right)}$	0.0123	0.0448	0.0527	$T_{22}$	$T_{n}^{\left(1\right)}$	0.0012	0.0127	0.0501
Empirical Powers, Significance Level α=0.05									
		Sample Size					Sample Size		
Model	Method	n=200	n=500	n=1000	Model	Method	n=200	n=500	n=1000
$T_{31}$	$T_{n}^{\left(1\right)}$	0.8414	0.9507	0.9972	$T_{32}$	$T_{n}^{\left(1\right)}$	0.5016	0.7979	0.967

**Table 7:** Empirical sizes and powers of $T_{n}^{\left(2\right)}$ and $T_{n}^{\left(3\right)}$ at level 0.05.

Empirical Sizes, Significance Level α=0.05									
		Sample Size					Sample Size		
Model	Method	n=200	n=500	n=1000	Model	Method	n=200	n=500	n=1000
$T_{41}$	$T_{n}^{\left(2\right)}$	0.0580	0.0466	0.0475	$T_{42}$	$T_{n}^{\left(2\right)}$	0.0320	0.0413	0.0445
	$T_{n}^{\left(3\right)}$	0.0504	0.0502	0.0495		$T_{n}^{\left(3\right)}$	0.0456	0.0487	0.0509
Empirical Power, Significance Level α=0.05									
		Sample Size					Sample Size		
Model	Method	n=200	n=500	n=1000	Model	Method	n=200	n=500	n=1000
$T_{51}$	$T_{n}^{\left(2\right)}$	0.9987	1.0000	1.0000	$T_{52}$	$T_{n}^{\left(2\right)}$	0.9987	1.0000	1.0000
	$T_{n}^{\left(3\right)}$	1.0000	1.0000	1.0000		$T_{n}^{\left(3\right)}$	1.0000	1.0000	1.0000

**Table 8:** Fitting results of different models: CML, SE, ${\hat{r}}_{CML}$, AIC, BIC, and RMS based on the first VOW dataset.

Model	Para.	CML	SE	${\hat{r}}_{\mathrm{CML}}$	AIC	BIC	RMS
SETINAR(2,1)	$\alpha_{1}$	0.0169	0.0221	6	1395.9685	1406.8836	3.3266
	$\alpha_{2}$	0.3071	0.0092				
	λ	5.9064	0.0032				
NBTINAR(1)	$\alpha_{1}$	0.2689	0.0034	6	1369.2936	1380.2087	12.3344
	$\alpha_{2}$	0.3371	0.0041				
	*v*	17.0000	0.0004				
RCTINAR(1)	$\phi_{1}$	0.0000	4.4123	6	1405.0371	1415.9521	3.3656
	$\phi_{2}$	0.2295	0.0034				
	λ	6.3869	0.0006				
BiNB−MTTINAR(1)	$\phi_{1}$	0.4852	0.0076	10	1358.6573	1369.5724	3.3655
(R = 0)	$\phi_{2}$	0.5202	0.0027				
	λ	3.7781	0.0013				
BiNB−MTTINAR(1)	$\phi_{1}$	0.3910	0.0038	4	1430.5887	1441.5038	3.7434
(R = 1)	$\phi_{2}$	0.6003	0.0012				
	λ	4.6621	0.0061				
TVMTTINAR(1)	$\beta_{1,0}$	−0.5391	−0.0006	6	**1352.1336**	1377.6020	3.1897
(R = 0)	$\beta_{1,1}$	0.0203	0.0001				
	$\beta_{1,2}$	0.0215	0.0000				
	$\beta_{2,0}$	0.0300	0.0001				
	$\beta_{2,1}$	−0.2134	−0.0005				
	$\beta_{2,2}$	0.0573	0.0003				
	λ	4.0801	0.0030				
TVMTTINAR(1)	$\beta_{1,0}$	−0.1093	−0.0002	12	1366.3313	1391.7998	3.2179
(R = 1)	$\beta_{1,1}$	−0.1298	−0.0003				
	$\beta_{1,2}$	0.0793	0.0002				
	$\beta_{2,0}$	−0.0765	−0.0002				
	$\beta_{2,1}$	−0.2039	−0.0007				
	$\beta_{2,2}$	0.0367	0.0002				
	λ	3.8956	0.0030				

**Table 9:** The other fitting results of the TVMTTINAR(1) model.

Rate	$T_{n}^{\left(1\right)}$	$T_{n}^{\left(3\right)}$	Mean(${\mathrm{Pr}}_{t}\left(\hat{\theta }\right)$)	Var(${\mathrm{Pr}}_{t}\left(\hat{\theta }\right)$)
0.5018	2.2343	35.2307	0.0041	1.1116

**Table 10:** Fitting results of different models: CML, SE, ${\hat{r}}_{CML}$, AIC, BIC, and RMS based on the second VOW dataset.

Model	Para.	CML	SE	${\hat{r}}_{\mathrm{CML}}$	AIC	BIC	RMS
SETINAR(2,1)	$\alpha_{1}$	0.2470	0.0028	6	1257.6000	1268.5151	2.5162
	$\alpha_{2}$	0.3511	0.0068				
	λ	4.2692	0.0053				
NBTINAR(1)	$\alpha_{1}$	0.1245	0.0172	6	1259.0943	1270.0094	33.9008
	$\alpha_{2}$	0.1605	0.0104				
	*v*	39.0000	0.0001				
RCTINAR(1)	$\phi_{1}$	0.0228	0.0079	6	1268.9508	1279.8659	2.5308
	$\phi_{2}$	0.2181	0.0017				
	λ	5.4232	0.0007				
BiNB−MTTINAR(1)	$\phi_{1}$	0.4788	0.0073	8	1259.9032	1270.8183	2.5413
(R = 0)	$\phi_{2}$	0.4955	0.0022				
	λ	3.1526	0.0010				
BiNB−MTTINAR(1)	$\phi_{1}$	0.4441	0.0022	4	1295.9314	1306.8464	2.9048
(R = 1)	$\phi_{2}$	0.6682	0.0018				
	λ	3.3134	0.0041				
TVMTTINAR(1)	$\beta_{1,0}$	−0.3448	−0.0003	6	1258.9090	1284.3775	2.5260
(R = 0)	$\beta_{1,1}$	0.0404	0.0000				
	$\beta_{1,2}$	0.0176	0.0000				
	$\beta_{2,0}$	−0.2200	−0.0002				
	$\beta_{2,1}$	0.0163	0.0000				
	$\beta_{2,2}$	−0.0080	0.0000				
	λ	3.4359	0.0063				
TVMTTINAR(1)	$\beta_{1,0}$	−1.1063	−0.0010	5	**1254.5145**	1279.9829	2.4856
(R = 1)	$\beta_{1,1}$	0.1417	0.0001				
	$\beta_{1,2}$	−0.0427	0.0000				
	$\beta_{2,0}$	−19.9998	−4.3550				
	$\beta_{2,1}$	−0.2643	−0.0002				
	$\beta_{2,2}$	3.3629	0.0030				
	λ	4.9869	0.0072				

**Table 11:** The other fitting results of the TVMTTINAR(1) model.

Rate	$T_{n}^{\left(1\right)}$	$T_{n}^{\left(3\right)}$	Mean(${\mathrm{Pr}}_{t}\left(\hat{\theta }\right)$)	Var(${\mathrm{Pr}}_{t}\left(\hat{\theta }\right)$)
0.5267	1.6682	63.9769	0.0015	1.0148

## Data Availability

Data are contained within the article.

## Appendix A

**Proof of Proposition** **1.** Denote $\phi_{\max,t}=\max\left\{\phi_{1,t},\phi_{2,t}\right\}$ for all *t*. To simplify the notation, denote $Z_{t}=\left(Z_{0},\ldots ,Z_{t}\right)$. Compute to see that, under the stationary distribution,

(A1) $$\begin{array}{cc} E(X_{t}|Z_{t})\; & =E\left[\left(\phi_{1,t}\circ X_{t-1}\right)I_{1,t}^{R}|Z_{t}\right]+E\left[\left(\phi_{2,t}*X_{t-1}\right)I_{2,t}^{R}|Z_{t}\right]+\lambda \\ \; & =E\left[E\left(\left(\phi_{1,t}\circ X_{t-1}\right)I_{1,t}^{R}|X_{t-1}\right)|Z_{t}\right]+E\left[E\left(\left(\phi_{2,t}*X_{t-1}\right)I_{2,t}^{R}|X_{t-1}\right)|Z_{t}\right]+\lambda \\ \; & \leq \phi_{\max,t}E\left(X_{t-1}|Z_{t}\right)+\lambda \\ \; & \leq \cdots \\ \; & \leq {\left(\phi_{\max,t}\right)}^{t}E\left(X_{0}|Z_{t}\right)+\lambda \sum_{i=0}^{t-1}{\left(\phi_{\max,t}\right)}^{i}\leq \infty , \end{array}$$

Similarly, we have

$$\begin{array}{cc} E(X_{t}^{2}|Z_{t})= & E[{\left(\phi_{1,t}\circ X_{t-1}\right)}^{2}I_{1,t}^{R}+{\left(\phi_{2,t}*X_{t-1}\right)}^{2}I_{2,t}^{R}+\varepsilon_{t}^{2}\\ \; & +2\left(\phi_{1,t}\circ X_{t-1}I_{1,t}^{R}\varepsilon_{t}+\phi_{2,t}*X_{t-1}I_{2,t}^{R}\varepsilon_{t}\right)|Z_{t}]\\ \; & \leq [\left(\phi_{1,t}-\phi_{1,t}^{2}+2\phi_{1,t}\lambda \right)E\left(X_{t-1}|Z_{t}\right)+\phi_{1,t}^{2}E\left(X_{t-1}^{2}|Z_{t}\right)\\ \; & +\left(\phi_{2,t}+\phi_{2,t}^{2}+2\phi_{2,t}\lambda \right)E\left(X_{t-1}|Z_{t}\right)+\phi_{2,t}^{2}E\left(X_{t-1}^{2}|Z_{t}\right)+\lambda +\lambda^{2}\\ \; & \leq u_{\max,t}E\left(X_{t-1}|Z_{t}\right)+\phi_{\max,t}^{2}E\left(X_{t-1}^{2}|Z_{t}\right)+\lambda +\lambda^{2} \end{array}$$

where $u_{\max,t}=\max\left\{\left(\phi_{1,t}-\phi_{1,t}^{2}+2\phi_{1,t}\lambda \right),\left(\phi_{2,t}+\phi_{2,t}^{2}+2\phi_{2,t}\lambda \right)\right\}$ for all *t*. If t=1, $E\left(X_{1}^{2}|Z_{t}\right)\leq u_{\max}E\left(X_{0}|Z_{t}\right)+\phi_{\max}^{2}E\left(X_{0}^{2}|Z_{t}\right)+\lambda +\lambda^{2}<\infty$. Else, if t≥2,

(A2) $$\begin{array}{cc} E(X_{t}^{2}|Z_{t}) & \leq \sum_{i=0}^{t-1}u_{\max}\phi_{\max}^{t-1+i}E\left(X_{0}|Z_{t}\right)+\lambda \sum_{i=0}^{t-2}u_{\max}\phi_{\max}^{t-2+i}+\left(\lambda +\lambda^{2}\right)\sum_{i=0}^{t-1}\phi_{\max}^{2i}\\ \; & <\infty . \end{array}$$

A similar, but tedious calculation shows that $E(X_{t}^{3}|Z_{t})\leq \infty$ and $E(X_{t}^{4}|Z_{t})\leq \infty$. Combining (A1) and (A2), one can see that $E(X_{t}^{k}|Z_{t})<\infty$ for k=1,2,3,4. □

**Proof of Section** **2.** The results $E\left(X_{t}|X_{t-1},Z_{t}\right),E\left(X_{t}|Z_{t}\right)$ and $\mathrm{Var}(X_{t}|X_{t-1},Z_{t})$ are straightforward to verify. We prove the other results of the moments and conditional moments: (1) The variance of $X_{t}$ under the condition $Z_{t}$ is given by

(A3) $$\begin{array}{cc} \mathrm{Var}(X_{t}|Z_{t})\; & =\mathrm{Var}\left[I_{1,t}^{R}\left(\phi_{1,t}\circ X_{t-1}\right)|Z_{t}\right]+\mathrm{Var}\left[I_{2,t}^{R}\left(\phi_{2,t}*X_{t-1}\right)|Z_{t}\right]\\ \; & +2\mathrm{Cov}(I_{1,t}^{R}\left(\phi_{1,t}\circ X_{t-1}\right),I_{2,t}^{R}\left(\phi_{2}*X_{t-1}\right)|Z_{t})+\lambda \\ \; & =I+II+III+\lambda . \end{array}$$

A direct calculation shows

(A4) $$\begin{array}{cc} I & =\mathrm{Var}\left\{E\left[I_{1,t}^{R}\left(\phi_{1}\circ X_{t-1}\right)|X_{t-1}\right]|Z_{t}\right\}+E\left\{\mathrm{Var}\left[I_{1,t}^{R}\left(\phi_{1}\circ X_{t-1}\right)|X_{t-1}\right]|Z_{t}\right\}\\ \; & =\phi_{1,t}^{2}\mathrm{Var}\left(I_{1,t}^{R}X_{t-1}\right)+\left(\phi_{1,t}\left(1-\phi_{1,t}\right)E\left(I_{1,t}^{R}X_{t-1}\right)\right)\\ \; & =\phi_{1,t}^{2}E\left(I_{1,t}^{R}X_{t-1}^{2}\right)-\phi_{1}^{2}p_{1}^{2}\mu_{1}^{2}+p_{1}\phi_{1,t}\left(1-\phi_{1,t}\right)\mu_{1}\\ \; & ={\left[\frac{\exp(Z_{t}^{⊤}\beta_{1})}{1+\exp(Z_{t}^{⊤}\beta_{1})}\right]}^{2}\left[p_{1}\left(\sigma_{1}^{2}+\mu_{1}^{2}\right)-p_{1}^{2}\mu_{1}^{2}\right]+\frac{\exp(Z_{t}^{⊤}\beta_{1})}{{\left[1+\exp\left(Z_{t}^{⊤}\beta_{1}\right)\right]}^{2}}p_{1}\mu_{1}. \end{array}$$

Similarly, we have

(A5) $$\begin{array}{cc} II & =\mathrm{Var}\left\{E\left[I_{2,t}^{R}\left(\phi_{2}*X_{t-1}\right)|X_{t-1}\right]|Z_{t}\right\}+E\left\{\mathrm{Var}\left[I_{2,t}^{R}\left(\phi_{2}*X_{t-1}\right)|X_{t-1}\right]|Z_{t}\right\}\\ \; & =\phi_{2,t}^{2}\mathrm{Var}\left(I_{2,t}^{R}X_{t-1}\right)+\left(\phi_{2,t}\left(1-\phi_{2,t}\right)E\left(I_{2,t}^{R}X_{t-1}\right)\right)\\ \; & =\phi_{2,t}^{2}E\left(I_{2,t}^{R}X_{t-1}^{2}\right)-\phi_{2}^{2}p_{2}^{2}\mu_{2}^{2}+p_{2}\phi_{2,t}\left(1+\phi_{2,t}\right)\mu_{2}\\ \; & ={\left[\frac{\exp(Z_{t}^{⊤}\beta_{2})}{1+\exp(Z_{t}^{⊤}\beta_{2})}\right]}^{2}\left[p_{2}\left(\sigma_{2}^{2}+\mu_{2}^{2}\right)-p_{2}^{2}\mu_{2}^{2}\right]+\frac{\exp\left(Z_{t}^{⊤}\beta_{2}\right)\left(2+\exp\left(Z_{t}^{⊤}\beta_{2}\right)\right)}{{\left[1+\exp\left(Z_{t}^{⊤}\beta_{2}\right)\right]}^{2}}p_{2}\mu_{2}. \end{array}$$

and

(A6) $$\begin{array}{cc} III & =2\mathrm{Cov}(I_{1,t}^{R}\left(\phi_{1,t}\circ X_{t-1}\right),I_{2,t}^{R}\left(\phi_{2}*X_{t-1}\right)|Z_{t})\\ \; & =-2\frac{\exp(Z_{t}^{⊤}\beta_{1})}{1+\exp(Z_{t}^{⊤}\beta_{1})}\frac{\exp(Z_{t}^{⊤}\beta_{2})}{1+\exp(Z_{t}^{⊤}\beta_{2})}p_{1}p_{2}\mu_{1}\mu_{2}. \end{array}$$

Then, $\mathrm{Var}(X_{t}|Z_{t})$ follows by replacing (A4), (A5) and (A6) in (A3) and some algebra. (2) The variance of $X_{t}$ is given by

(A7) $$\begin{array}{cc} \mathrm{Var}(X_{t})\; & =\mathrm{Var}\left[I_{1,t}^{R}\left(\phi_{1,t}\circ X_{t-1}\right)\right]+\mathrm{Var}\left[I_{2,t}^{R}\left(\phi_{2,t}*X_{t-1}\right)\right]\\ \; & +2\mathrm{Cov}(I_{1,t}^{R}\left(\phi_{1,t}\circ X_{t-1}\right),I_{2,t}^{R}\left(\phi_{2}*X_{t-1}\right))+\lambda \\ \; & =I+II+III+\lambda . \end{array}$$

Similar to the derivation of $\mathrm{Var}(X_{t}|Z_{t})$,

(A8) $$\begin{array}{cc} I & =\mathrm{Var}\left\{E\left[I_{1,t}^{R}\left(\phi_{1}\circ X_{t-1}\right)|X_{t-1},Z_{t}\right]\right\}+E\left\{\mathrm{Var}\left[I_{1,t}^{R}\left(\phi_{1}\circ X_{t-1}\right)|X_{t-1},Z_{t}\right]\right\}\\ \; & =\mathrm{Var}\left[I_{1,t}^{R}\int f\left(\phi_{1,t}\right)\phi_{1,t}X_{t-1}d\phi_{1,t}\right]+E\left\{E\left[I_{1,t}^{R}\phi_{1,t}\left(1-\phi_{1,t}\right)X_{t-1}\right]+\mathrm{Var}\left(I_{1,t}^{R}\phi_{1,t}X_{t-1}\right)\right\}\\ \; & =\mathrm{Var}\left[I_{1,t}^{R}X_{t-1}E\left(\phi_{1,t}\right)\right]+E\left\{I_{1,t}^{R}X_{t-1}E\left[\phi_{1,t}\left(1-\phi_{1,t}\right)\right]+I_{1,t}^{R}X_{t-1}\mathrm{Var}\left(\phi_{1,t}\right)\right\}\\ \; & =\phi_{1}^{2}\left[p_{1}\left(\sigma_{1}^{2}+\mu_{1}^{2}\right)-p_{1}^{2}\mu_{1}^{2}\right]+p_{1}\mu_{1}\left(\phi_{1}-\sigma_{\phi_{1}}^{2}-\phi_{1}^{2}\right)+p_{1}\sigma_{\phi_{1}}^{2}\left(\sigma_{1}^{2}+\mu_{1}^{2}\right). \end{array}$$

Similarly, we have

(A9) $$\begin{array}{cc} II & =\mathrm{Var}\left\{E\left[I_{2,t}^{R}\left(\phi_{2=}\circ X_{t-1}\right)|X_{t-1},Z_{t}\right]\right\}+E\left\{\mathrm{Var}\left[I_{2,t}^{R}\left(\phi_{2}\circ X_{t-1}\right)|X_{t-1},Z_{t}\right]\right\}\\ \; & =\mathrm{Var}\left[I_{2,t}^{R}\int f\left(\phi_{2,t}\right)\phi_{2,t}X_{t-1}d\phi_{2,t}\right]+E\left\{E\left[I_{1,t}^{R}\phi_{2,t}\left(1+\phi_{2,t}\right)X_{t-1}\right]+\mathrm{Var}\left(I_{2,t}^{R}\phi_{2,t}X_{t-1}\right)\right\}\\ \; & =\mathrm{Var}\left[I_{2,t}^{R}X_{t-1}E\left(\phi_{2,t}\right)\right]+E\left\{I_{2,t}^{R}X_{t-1}E\left[\phi_{2,t}\left(1+\phi_{2,t}\right)\right]+I_{2,t}^{R}X_{t-1}\mathrm{Var}\left(\phi_{2,t}\right)\right\}\\ \; & =\phi_{2}^{2}\left[p_{2}\left(\sigma_{2}^{2}+\mu_{2}^{2}\right)-p_{2}^{2}\mu_{2}^{2}\right]+p_{2}\mu_{2}\left(\phi_{2}+\sigma_{\phi_{2}}^{2}+\phi_{2}^{2}\right)+p_{2}\sigma_{\phi_{2}}^{2}\left(\sigma_{2}^{2}+\mu_{2}^{2}\right). \end{array}$$

and

(A10) $$\begin{array}{cc} III & =2\mathrm{Cov}(I_{1,t}^{R}\left(\phi_{1,t}\circ X_{t-1}\right),I_{2,t}^{R}\left(\phi_{2}*X_{t-1}\right))\\ \; & =-2\phi_{1}\phi_{2}p_{1}p_{2}\mu_{1}\mu_{2}. \end{array}$$

Then, $\mathrm{Var}(X_{t})$ follows by replacing (A8)–(A10) in (A7) and some algebra. (3) For the conditional autocovariance $\mathrm{Cov}(X_{t},X_{t+h}|Z_{t+1},\ldots ,Z_{t+h})$, when h=1,

$$\begin{array}{cc} \mathrm{Cov}(X_{t},X_{t+1}|Z_{t+1}) & =\mathrm{Cov}[X_{t},E\left(X_{t+1}|X_{t}\right)|Z_{t+1}]\\ \; & =\mathrm{Cov}\{X_{t},\left[\phi_{1,t+1}X_{t}+\lambda \right]I_{1,t+1}^{R}+\left[\phi_{2,t+1}X_{t}+\lambda \right]I_{2,t+1}^{R}|Z_{t+1}\}\\ \; & =\sum_{i=1}^{2}\left[\phi_{i,t+1}\mathrm{Cov}\left(X_{t},I_{i,t+1}^{R}X_{t}\right)\right], \end{array}$$

where

$$\begin{array}{cc} \mathrm{Cov}(X_{t},I_{i,t+1}^{R}X_{t}) & =E\left(I_{i,t+1}^{R}X_{t}X_{t}\right)-E\left(I_{i,t+1}^{R}X_{t}\right)E\left(X_{t}\right)=p_{i}\left(\sigma_{i}^{2}+\mu_{i}^{2}\right)-p_{i}\mu_{i}E\left(X_{t}\right), \end{array}$$

Then,

$$\begin{array}{c} \mathrm{Cov}\left(X_{t},X_{t+1}\right)=\sum_{i=1}^{2}\left\{\phi_{i}p_{i}\left[\left(\sigma_{i}^{2}+\mu_{i}^{2}\right)-\mu_{i}E\left(X_{t}\right)\right]\right\}=\sum_{i=1}^{2}\phi_{i}p_{i}\gamma_{0}^{\left(i\right)}. \end{array}$$

When h>1, there is

$$\begin{array}{cc} \mathrm{Cov}(X_{t},X_{t+h}|Z_{t+h}) & =\mathrm{Cov}[X_{t},E\left(X_{t+h}|X_{t+h-1}\right)|Z_{t+h}]\\ \; & =\mathrm{Cov}\{X_{t},\left(\phi_{1,t+h}X_{t+h-1}\right)I_{1,t+h}^{R}+\left(\phi_{2,t+h}X_{t+h-1}\right)I_{2,t+h}^{R}+\lambda |Z_{t+h}\}\\ \; & =\sum_{i=1}^{2}\phi_{i,t+h}\left[\mathrm{Cov}\left(X_{t},I_{i,t+h}^{R}X_{t+h-1}\right)\right],\\ \; & =\sum_{i=1}^{2}\frac{\exp(Z_{t+h}^{⊤}\beta_{i})}{1+\exp(Z_{t+h}^{⊤}\beta_{i})}\mathrm{Cov}\left(X_{t},I_{i,t+h}^{R}X_{t+h-1}\right), \end{array}$$

where

$$\begin{array}{cc} \mathrm{Cov}(X_{t},I_{i,t+h}^{R}X_{t+h-1}) & =p_{i}\mathrm{Cov}\left(X_{t},X_{t+h-1}|X_{t+h-1}\leq r\right)=p_{i}\gamma_{h-1}^{\left(i\right)}, \end{array}$$

Then, we obtain

$$\begin{array}{c} \mathrm{Cov}\left(X_{t},X_{t+h}\right)=\sum_{i=1}^{2}\frac{\exp(Z_{t+h}^{⊤}\beta_{i})}{1+\exp(Z_{t+h}^{⊤}\beta_{i})}p_{i}\gamma_{h-1}^{\left(i\right)}. \end{array}$$

(4) For the unconditional autocovariance $\mathrm{Cov}(X_{t},X_{t+h})$, when h=1,

$$\begin{array}{cc} \mathrm{Cov}(X_{t},X_{t+1}|Z_{t+1}) & =\mathrm{Cov}[X_{t},E\left(X_{t+1}|X_{t}\right)]\\ \; & =\mathrm{Cov}\{X_{t},\left(\phi_{1,t+1}X_{t}\right)I_{1,t+1}^{R}+\left(\phi_{2,t+1}X_{t}\right)I_{2,t+1}^{R}+\lambda \}\\ \; & =\sum_{i=1}^{2}\mathrm{Cov}\left(X_{t},\left[\phi_{i,t+1}I_{i,t+1}^{R}X_{t}\right)\right], \end{array}$$

where

$$\begin{array}{cc} \mathrm{Cov}(X_{t},\phi_{i,t+1}I_{i,t+1}^{R}X_{t}) & =E\left(\phi_{i,t+1}I_{i,t+1}^{R}X_{t}X_{t}\right)-E\left(\phi_{i,t+1}I_{i,t+1}^{R}X_{t}\right)E\left(X_{t}\right)\\ \; & =\mu_{\phi_{i}}^{\left(1\right)}p_{i}\left(\sigma_{i}^{2}+\mu_{i}^{2}\right)-\mu_{\phi_{i}}^{\left(1\right)}p_{i}\mu_{i}E\left(X_{t}\right), \end{array}$$

Then,

$$\begin{array}{c} \mathrm{Cov}\left(X_{t},X_{t+1}\right)=\sum_{i=1}^{2}\left\{\mu_{\phi_{i}}^{\left(1\right)}p_{i}\left[\left(\sigma_{i}^{2}+\mu_{i}^{2}\right)-\mu_{i}E\left(X_{t}\right)\right]\right\}=\sum_{i=1}^{2}\mu_{\phi_{i}}^{\left(1\right)}p_{i}\gamma_{0}^{\left(i\right)}. \end{array}$$

When h>1, there is

$$\begin{array}{cc} \mathrm{Cov}(X_{t},X_{t+h}) & =\mathrm{Cov}[X_{t},E\left(X_{t+h}|X_{t+h-1}\right)]\\ \; & =\mathrm{Cov}\{X_{t},\left(\phi_{1,t+h}X_{t+h-1}\right)I_{1,t+h}^{R}+\left(\phi_{2,t+h}X_{t+h-1}\right)I_{2,t+h}^{R}+\lambda |Z_{t+h}\}\\ \; & =\sum_{i=1}^{2}\left[\mathrm{Cov}\left(X_{t},\phi_{i,t+h}I_{i,t+h}^{R}X_{t+h-1}\right)\right], \end{array}$$

where

$$\begin{array}{cc} \mathrm{Cov}(X_{t},\phi_{i,t+h}I_{i,t+h}^{R}X_{t+h-1}) & =\mu_{\phi_{i}}^{\left(h\right)}p_{i}\mathrm{Cov}\left(X_{t},X_{t+h-1}|X_{t+h-1}\leq r\right)=\mu_{\phi_{i}}^{\left(h\right)}p_{i}\gamma_{h-1}^{\left(i\right)}, \end{array}$$

Then, we obtain

$$\begin{array}{c} \mathrm{Cov}\left(X_{t},X_{t+h}\right)=\sum_{i=1}^{2}\mu_{\phi_{i}}^{\left(h\right)}p_{i}\gamma_{h-1}^{\left(i\right)}. \end{array}$$

Thus, the autocorrelation function $\rho \left(h\right)=\left[\sum_{i=1}^{2}\mu_{\phi_{i}}^{\left(h\right)}\phi_{i}p_{i}\gamma_{h-1}^{\left(i\right)}\right]\setminus \mathrm{Var}\left(X_{t}\right)$. □

**Proof of Theorem** **1.** By Taylor’s expansion, there is

$$\begin{array}{cc} 0 & =\frac{1}{\sqrt{n}}\sum_{t=1}^{n}-\frac{1}{2}\frac{\partial U_{t}^{2}\left({\hat{\theta }}_{CLS}\right)}{\partial \theta }\\ \; & =\frac{1}{\sqrt{n}}\sum_{t=1}^{n}-\frac{1}{2}\frac{\partial U_{t}^{2}\left(\theta_{0}\right)}{\partial \theta }+\left(\frac{1}{n}\sum_{t=1}^{n}-\frac{1}{2}\frac{\partial^{2}U_{t}^{2}\left(\theta_{0}\right)}{\partial \theta \partial \theta^{⊤}}\right)\sqrt{n}\left({\hat{\theta }}_{CLS}-\theta_{0}\right)+o_{p}\left(n^{-1/2}\right). \end{array}$$

We first prove

$$\begin{array}{c} \frac{1}{\sqrt{n}}\sum_{t=1}^{n}-\frac{1}{2}\frac{\partial U_{t}^{2}\left({\hat{\theta }}_{CLS}\right)}{\partial \theta }\overset{d}{⟶}N\left(0,W\right). \end{array}$$

Now, let $F_{n}=\sigma \left\{X_{0},X_{1},\ldots ,X_{n},Z_{1},\ldots ,Z_{n+1}\right\}$, and for i=1,2,

$$\begin{array}{c} M_{n}^{\left(i,0\right)}=\sum_{t=1}^{n}-\frac{1}{2}\frac{\partial U_{t}^{2}\left(\theta_{0}\right)}{\partial \beta_{i,0}}=\sum_{t=1}^{n}\left[U_{t}\left(\theta_{0}\right)X_{t-1}I_{i,t}^{R}\frac{\exp(Z_{t}^{⊤}\beta_{i})}{{\left[1+\exp\left(Z_{t}^{⊤}\beta_{i}\right)\right]}^{2}}\right]. \end{array}$$

Then, we have

$$\begin{array}{cc} E(M_{n}^{\left(i,0\right)}|F_{n-1})\; & =E\{M_{n-1}^{\left(i,0\right)}+U_{n}\left(\theta_{0}\right)X_{n-1}I_{i,n}^{R}\frac{\exp(Z_{n}^{⊤}\beta_{i})}{{\left[1+\exp\left(Z_{n}^{⊤}\beta_{i}\right)\right]}^{2}}|F_{n-1}\}\\ \; & =M_{n-1}^{\left(i,0\right)}+E\left\{U_{n}\left(\theta_{0}\right)X_{n-1}I_{i,n}^{R}\frac{\exp(Z_{n}^{⊤}\beta_{i})}{{\left[1+\exp\left(Z_{n}^{⊤}\beta_{i}\right)\right]}^{2}}|F_{n-1}\right\}=M_{n-1}^{\left(i,0\right)}, \end{array}$$

i.e., $\left\{M_{n}^{\left(i,0\right)},F_{n},n⩾0\right\}$ is a martingale. By Proposition (1), $E(X_{t}^{4}|Z_{t})<\infty$, there is

$$E({\left(U_{t}\left(\theta_{0}\right)\right)}^{2}X_{t-1}^{2}I_{i,n}^{R}{\left(\frac{\exp(Z_{t}^{⊤}\beta_{i})}{{\left[1+\exp\left(Z_{t}^{⊤}\beta_{i}\right)\right]}^{2}}\right)}^{2}|F_{t-1})<\infty .$$

Then, by the ergodic theorem, we have

$$\begin{array}{cc} \frac{1}{n}\sum_{t=2}^{n}({\left(U_{t}\left(\theta_{0}\right)\right)}^{2}X_{t-1}^{2}I_{i,n}^{R}{\left(\frac{\exp(Z_{t}^{⊤}\beta_{i})}{{\left[1+\exp\left(Z_{t}^{⊤}\beta_{i}\right)\right]}^{2}}\right)}^{2}\overset{a.s.}{⟶} & E{\left[U_{t}\left(\theta_{0}\right)X_{t-1}I_{i,n}^{R}\frac{\exp(Z_{t}^{⊤}\beta_{i})}{{\left[1+\exp\left(Z_{t}^{⊤}\beta_{i}\right)\right]}^{2}}\right]}^{2}\\ \; & =E\left[U_{t}^{2}\left(\theta_{0}\right){\left(\frac{\partial }{\partial \beta_{i,0}}g\left(\theta_{0},X_{t-1},Z_{t}\right)\right)}^{2}\right]\\ \; & =W_{(i-1)q+i,(i-1)q+i}, \end{array}$$

Thus, by Corollary 3.2 from [23], the martingale central limit theorem applies, and we obtain $M_{n}^{\left(i,0\right)}/\sqrt{n}\overset{d}{⟶}N\left(0,W_{(i-1)q+i,(i-1)q+i}\right).$ Similarly, we can prove that, for any i=1,2,s=1,…,q,

$$\begin{array}{c} M_{n}^{\left(i,s\right)}=\sum_{t=1}^{n}-\frac{1}{2}\frac{\partial U_{t}^{2}\left(\theta_{0}\right)}{\partial \beta_{i,s}}=\sum_{t=1}^{n}\left[U_{t}\left(\theta_{0}\right)X_{t-1}I_{i,t}^{R}\frac{Z_{s,t}\exp\left(Z_{t}^{⊤}\beta_{i}\right)}{{\left[1+\exp\left(Z_{t}^{⊤}\beta_{i}\right)\right]}^{2}}\right]. \end{array}$$

is a martingale, and we have that

$$\begin{array}{cc} \frac{1}{n}\sum_{t=1}^{n}[U_{t}\left(\theta_{0}\right)X_{t-1}I_{i,t}^{R}\frac{Z_{s,t}\exp\left(Z_{t}^{⊤}\beta_{i}\right)}{{\left[1+\exp\left(Z_{t}^{⊤}\beta_{i}\right)\right]}^{2}}{]}^{2}\overset{a.s.}{⟶} & E[U_{t}\left(\theta_{0}\right)X_{t-1}I_{i,t}^{R}\frac{Z_{s,t}\exp\left(Z_{t}^{⊤}\beta_{i}\right)}{{\left[1+\exp\left(Z_{t}^{⊤}\beta_{i}\right)\right]}^{2}}{]}^{2}\\ \; & =E\left[U_{t}^{2}\left(\theta_{0}\right){\left(\frac{\partial }{\partial \beta_{i,s}}g\left(\theta_{0},X_{t-1},Z_{t}\right)\right)}^{2}\right]\\ \; & =W_{(i-1)q+i+s,(i-1)q+i+s}, \end{array}$$

that is $M_{n}^{\left(i,s\right)}/\sqrt{n}\overset{d}{⟶}N\left(0,W_{(i-1)q+i+s,(i-1)q+i+s}\right).$ Furthermore, we can also prove that

$$\begin{array}{c} M_{n}^{\left(2q+3\right)}=\sum_{t=1}^{n}-\frac{1}{2}\frac{\partial U_{t}^{2}\left(\theta_{0}\right)}{\partial \lambda }=\sum_{t=1}^{n}U_{t}\left(\theta_{0}\right). \end{array}$$

is a martingale and

$$\begin{array}{c} \frac{1}{n}\sum_{t=1}^{n}{\left[U_{t}\left(\theta_{0}\right)\right]}^{2}\overset{a.s.}{⟶}EU_{t}^{2}\left(\theta_{0}\right)=W_{2q+3,2q+3}, \end{array}$$

that is $M_{n}^{\left(2q+3\right)}/\sqrt{n}\overset{d}{⟶}N\left(0,W_{2q+3,2q+3}\right).$ In the same way, for any $c={\left(c_{1},\ldots ,c_{2q+3}\right)}^{⊤}\in R^{2q+3}\setminus 0_{2q+3,1}$, we have

$$\begin{array}{cc} \; & \frac{1}{\sqrt{n}}c^{⊤}{\left(M_{n}^{\left(1,0\right)},\ldots M_{n}^{\left(1,q\right)},M_{n}^{\left(2,0\right)},\ldots ,M_{n}^{\left(2,q\right)},M_{n}^{\left(2q+3\right)}\right)}^{⊤}\\ \; & =\frac{1}{\sqrt{n}}\sum_{t=1}^{n}U_{t}\left(\theta_{0}\right)\left\{\sum_{i=1}^{2}\left[\sum_{s=0}^{q}c_{(i-1)q+s+i}X_{t-1}\frac{Z_{s,t}\exp\left(Z_{t}^{⊤}\beta_{i}\right)}{{\left[1+\exp\left(Z_{t}^{⊤}\beta_{i}\right)\right]}^{2}}\right]I_{i,t}^{R}+c_{2q+3}\right\}\\ \; & \overset{d}{⟶}N\left(0,E{\left\{U_{t}\left(\theta_{0}\right)\left\{\sum_{i=1}^{2}\left[\sum_{s=0}^{q}c_{(i-1)q+s+i}X_{t-1}\frac{Z_{s,t}\exp\left(Z_{t}^{⊤}\beta_{i}\right)}{{\left[1+\exp\left(Z_{t}^{⊤}\beta_{i}\right)\right]}^{2}}\right]I_{i,t}^{R}+c_{2q+3}\right\}\right\}}^{2}\right). \end{array}$$

Thus, by the Cramér–Wold device,

$$\begin{array}{cc} \frac{1}{\sqrt{n}}\sum_{t=1}^{n}-\frac{1}{2}\frac{\partial U_{t}^{2}\left(\theta_{0}\right)}{\partial \theta }=\frac{1}{\sqrt{n}}{\left(M_{n}^{\left(1,0\right)},\ldots M_{n}^{\left(1,q\right)},M_{n}^{\left(2,0\right)},\ldots ,M_{n}^{\left(2,q\right)},M_{n}^{\left(2q+3\right)}\right)}^{⊤}\; & \overset{d}{⟶}N\left(0,W\right), \end{array}$$

Consider the second item of Taylor’s expansion, $\frac{1}{n}\sum_{t=1}^{n}-\frac{1}{2}\frac{\partial^{2}U_{t}^{2}\left(\theta_{0}\right)}{\partial \theta \partial \theta^{⊤}}$:

$$\begin{array}{cc} \; & \frac{1}{n}\sum_{t=1}^{n}-\frac{1}{2}\frac{\partial^{2}U_{t}^{2}\left(\theta_{0}\right)}{\partial \theta \partial \theta^{⊤}}\\ \; & =\frac{1}{n}\sum_{t=1}^{n}\left(-\frac{\partial g(\theta_{0},X_{t-1},Z_{t})}{\partial \theta^{⊤}}\frac{\partial g(\theta_{0},X_{t-1},Z_{t})}{\partial \theta }+\frac{\partial^{2}g\left(\theta_{0},X_{t-1},Z_{t}\right)}{\partial \theta \partial \theta^{⊤}}\left(X_{t}-g\left(\theta_{0},X_{t-1},Z_{t}\right)\right)\right). \end{array}$$

Note that

$$E(\frac{\partial^{2}g\left(\theta_{0},X_{t-1},Z_{t}\right)}{\partial \theta \partial \theta^{⊤}}\left(X_{t}-g\left(\theta_{0},X_{t-1},Z_{t}\right)\right))=0$$

and then, by the ergodic theorem, we have

$$\begin{array}{c} \lim_{n\to \infty }\frac{1}{n}\sum_{t=1}^{n}-\frac{1}{2}\frac{\partial^{2}U_{t}^{2}\left(\theta_{0}\right)}{\partial \theta \partial \theta^{⊤}}=-V. \end{array}$$

Hence, we have that

$$\begin{array}{c} \sqrt{n}\left({\hat{\theta }}_{CLS}-\theta_{0}\right)\overset{d}{⟶}N\left(0,V^{-1}WV^{-1}\right), \end{array}$$

and the proof has been completed. □

**Proof of Theorem** **2.** Considering that the case R=0 is similar to R=1, we only prove the case R=0. We first prove the consistency of ${\hat{\theta }}_{CML}$. See [24] for a similar technique. Clearly, L(θ) is a measurable function of $x_{t}$ for all $\theta \in \Theta_{\theta }$; it is continuous in an open and convex neighborhood $N(\theta_{0})$. From the assumption in Theorem 2, it ensures that $E[\ell_{t}\left(\theta \right)]$ has a unique maximizer in the compact set $\Theta_{\theta }$, say $\overset{ˇ}{\theta }$. We can assume $\overset{ˇ}{\theta }\in N\left(\theta_{0}\right)$. Meanwhile, for arbitrary point $\theta \in N(\theta_{0})$, by Jensen’s inequality, we have

(A11) $$\begin{array}{c} E\left[\ell_{t}\left(\theta \right)-\ell_{t}\left(\theta_{0}\right)\right]=E\left[\log\left(\frac{P_{\theta }\left(z_{t},X_{t-1},X_{t}\right)}{P_{\theta_{0}}\left(z_{t},X_{t-1},X_{t}\right)}\right)\right]⩽\log\left[E\left(\frac{P_{\theta }\left(z_{t},X_{t-1},X_{t}\right)}{P_{\theta_{0}}\left(z_{t},X_{t-1},X_{t}\right)}\right)\right]=0. \end{array}$$

That is, $E[\ell_{t}\left(\theta \right)]$ is a strict local maximum at $\theta_{0}$. In the following, we will show that the log-likelihood function $\sum_{t=1}^{n}\ell_{t}\left(\theta \right)/n$ converges almost surely and uniformly in $\Theta_{\theta }$ to $E[\ell_{t}\left(\theta \right)]$, that is

$$\begin{array}{c} {\left|\left|\frac{1}{n}\sum_{t=1}^{n}\ell_{t}\left(\theta \right)-E\left[\ell_{t}\left(\theta \right)\right]\right|\right|}_{\Theta_{\theta }}\overset{a.s.}{\to }0, \end{array}$$

as n→∞. Then, the almost sure convergence of ${\hat{\theta }}_{CML}$ such that ${\hat{\theta }}_{CML}\overset{a.s.}{\to }\theta_{0}$ follows by standard arguments due to [25]. Note that $\left\{X_{t}\right\}$ is stationary and ergodic, then $\ell_{t}\left(\theta \right)$ is a stationary and ergodic sequence of random elements that take values in the space of continuous functions $C(\Theta_{\theta },R)$ equipped with the uniform norm $\left|\right|\cdot {\left|\right|}_{\Theta_{\theta }}$. Therefore, the desired convergence result follows by an application of the ergodic theorem of [26], if the uniform integrability condition $E||\ell_{t}\left(\theta \right){\left|\right|}_{\Theta_{\theta }}<\infty$ is satisfied. Note that

|ℓt(θ)|=−log(I1,tR∑m=0min(xt−1,xt)xt−1me−λλxt−m(xt−m)!ϕ1,tm(1−ϕ1,t)xt−1−m+I2,tR∑m=0xtΓ(xt−1+m)Γ(xt−1)Γ(m+1)ϕ2,tm(1+ϕ2,t)xt−1+me−λλxt−m(xt−m)!).

For convenience, we first assume that $x_{t-1}⩽r$ and $x_{t-1}<x_{t}$, i.e., $(x_{t}-x_{t-1})⩾1$, then together with log(x)<x, there is

$$\begin{array}{cc} |\ell_{t}\left(\theta \right)| & ⩽-\log\left(e^{-\lambda }\frac{\lambda^{x_{t}-x_{t-1}}}{(x_{t}-x_{t-1})!}\phi_{1,t}^{x_{t-1}}\right)\\ \; & ⩽-\left(-\lambda +\left(x_{t}-x_{t-1}\right)\log\left(\lambda \right)-\log\left[\left(x_{t}-x_{t-1}\right)\right]+x_{t-1}\log\left(\phi_{1,t}\right)\right)\\ \; & ⩽\left(\lambda -\left(x_{t}-x_{t-1}\right)\log\left(\lambda \right)+\left(x_{t}-x_{t-1}\right)-x_{t-1}\log\left(\phi_{1,t}\right)\right)\\ \; & ⩽x_{t}\left[1-\log\left(\lambda \right)\right]-x_{t-1}\left[\log\left(\phi_{1,t}\right)-\log\left(\lambda \right)+1\right]+\lambda \end{array}$$

almost surely for any $\theta \in \Theta_{\theta }$. Similarly, if $x_{t-1}=x_{t}$ and we assume $x_{t-1}⩽r$,

$$\begin{array}{c} |\ell_{t}\left(\theta \right)|⩽\lambda -x_{t}\log\phi_{1,t} \end{array}$$

almost surely for any $\theta \in \Theta_{\theta }$. If $x_{t-1}>x_{t}$ and we assume $x_{t-1}⩽r$,

|ℓt(θ)|⩽−logxt−1xte−λϕ1,txt(1−ϕ1,t)xt−1−xt⩽∑j=1xt−1−xtlogj−∑j=1xt−1logj+λ+xt{log[(1−ϕ1,t)]−log[ϕ1,t]}−xt−1log[(1−ϕ1,t)]⩽λ+xt{log[(1−ϕ1,t)]−log[ϕ1,t]}−xt−1log[(1−ϕ1,t)]

almost surely for any $\theta \in \Theta_{\theta }$. Analogously, in terms of $x_{t-1}>r$, there is

$$\begin{array}{c} |\ell_{t}\left(\theta \right)|⩽\lambda +x_{t}\left\{\log\left[\left(1-\phi_{2,t}\right)\right]-\log\left[\phi_{2,t}\right]\right\}+x_{t-1}\log\left[\left(1+\phi_{1,t}\right)\right] \end{array}$$

almost surely for any $\theta \in \Theta_{\theta }$. Clearly, $E(X_{t})<\infty$. We have the conclusion $E\left|\right|\ell_{t}\left(\theta \right){\left|\right|}_{\Theta_{\theta }}<\infty$, and the strong consistency has been proven. To prove this asymptotically, we study the method in [15] and perform the Taylor expansion of the score vector around $\theta_{0}$:

$$\begin{array}{c} 0=\frac{1}{\sqrt{n}}\frac{\partial L({\hat{\theta }}_{CML})}{\partial \theta }=\frac{1}{\sqrt{n}}\frac{\partial L(\theta_{0})}{\partial \theta }+\left(\frac{1}{n}\frac{\partial^{2}L(\theta_{n}^{*})}{\partial \theta \partial \theta^{⊤}}\right)\sqrt{n}\left({\hat{\theta }}_{CML}-\theta_{0}\right), \end{array}$$

where $\theta_{n}^{*}$ lies in between ${\hat{\theta }}_{CML}$ and $\theta_{0}$. It is easy to see $E{\left(\frac{\partial \ell_{t}\left(\theta \right)}{\partial \theta }\right)}_{\theta_{0}}=0$, which implies that $\left\{\ell_{t}\left(\theta_{0}\right)/\partial \theta \right\}$ is a martingale difference, and

$$\mathrm{Cov}\left(\frac{\partial \ell_{t}\left(\theta_{0}\right)}{\partial \theta }\right)=E{\left[\left(\frac{\partial \ell_{t}\left(\theta \right)}{\partial \theta }\right){\left(\frac{\partial \ell_{t}\left(\theta \right)}{\partial \theta }\right)}^{⊤}\right]}_{\theta_{0}}.$$

By the Cramér–Wold device and the central limit theorem in Theorem 18.3 of Billingsley [27], it follows that

$$\begin{array}{c} \frac{1}{\sqrt{n}}\frac{\partial L(\theta_{0})}{\partial \theta }\overset{d}{⟶}N\left(0,I(\theta_{0})\right)\;\mathrm{with}\;I\left(\theta_{0}\right)=E{\left[\frac{\partial \ell_{t}\left(\theta \right)}{\partial \theta }\frac{\partial \ell_{t}\left(\theta \right)}{\partial \theta^{⊤}}\right]}_{\theta_{0}}. \end{array}$$

Next, we aim to show that $\frac{1}{n}\frac{\partial^{2}\ell (\theta_{n}^{*})}{\partial \theta \partial \theta^{⊤}}$ converges to a finite nonsingular matrix:

$$J\left(\theta_{0}\right)=E{\left(\frac{\partial^{2}\ell_{t}\left(\theta \right)}{\partial \theta \partial \theta^{⊤}}\right)}_{\theta_{0}}$$

for any $\theta_{n}^{*}\overset{a.s.}{\to }\theta_{0}$, where $\theta_{n}^{*}$ lies in between $\theta_{0}$ and ${\hat{\theta }}_{CML}$. Note that, for any i,j=1,…,2q+3, the Taylor’s expansion of the second-order derivatives of $L(\theta_{n}^{*})$ at $\theta_{0}$ is

$$\begin{array}{c} \frac{1}{n}\sum_{t=1}^{n}\frac{\partial^{2}l_{t}\left(\theta_{n}^{*}\right)}{\partial \theta_{i}\partial \theta_{j}}=\frac{1}{n}\sum_{t=1}^{n}\frac{\partial^{2}l_{t}\left(\theta_{0}\right)}{\partial \theta_{i}\partial \theta_{j}}+\left(\frac{1}{n}\sum_{t=1}^{n}\frac{\partial^{3}l_{t}\left(\tilde{\theta }\right)}{\partial \theta_{i}\partial \theta_{j}\partial \theta^{⊤}}\right)\left(\tilde{\theta }-\theta_{0}\right), \end{array}$$

where $\tilde{\theta }$ is between $\theta_{0}$ and $\theta_{n}^{*}$. According to the assumption in Theorem 2, there is

$$\bar{\lim_{n\to \infty }}\underset{\theta \in N(\theta_{0})}{\sup}|\frac{\partial^{3}\ell_{t}\left(\theta \right)}{\partial \theta_{i}\partial \theta_{j}\partial \theta_{k}}|<\infty ,$$

for any i,j,k=1,…,2q+3. Thus, combining the assumption $E{\left(\frac{1}{n}\frac{\partial^{2}\ell \left(\theta \right)}{\partial \theta \partial \theta^{⊤}}\right)}_{\theta_{0}}$ is a nonsingular matrix, the strong consistency of the estimates, and the ergodic theorem in [28], it is easy to obtain a conclusion:

$$\begin{array}{c} \frac{1}{n}\frac{\partial^{2}\ell (\theta_{n}^{*})}{\partial \theta \partial \theta^{⊤}}\overset{a.s.}{⟶}J\left(\theta_{0}\right), \end{array}$$

as n→∞. To sum up, there is

$$\begin{array}{c} \sqrt{n}\left({\hat{\theta }}_{CML}-\theta_{0}\right)\overset{d}{⟶}N\left(0,J^{-1}\left(\theta_{0}\right)I\left(\theta_{0}\right)J^{-1}\left(\theta_{0}\right)\right). \end{array}$$

as n→∞, and the proof has been completed. □
